# Supplementary figures and images for: Insights for disease modeling from single-cell transcriptomics of iPSC-derived Ngn2-induced neurons and astrocytes across differentiation time and co-culture
Source: BMC Biol. 2024 Apr 2;22:75. doi: 10.1186/s12915-024-01867-4 (PMC10985965; doi:10.1186/s12915-024-01867-4)

# Supplementary Figure 1: Astrocyte genes from table 1

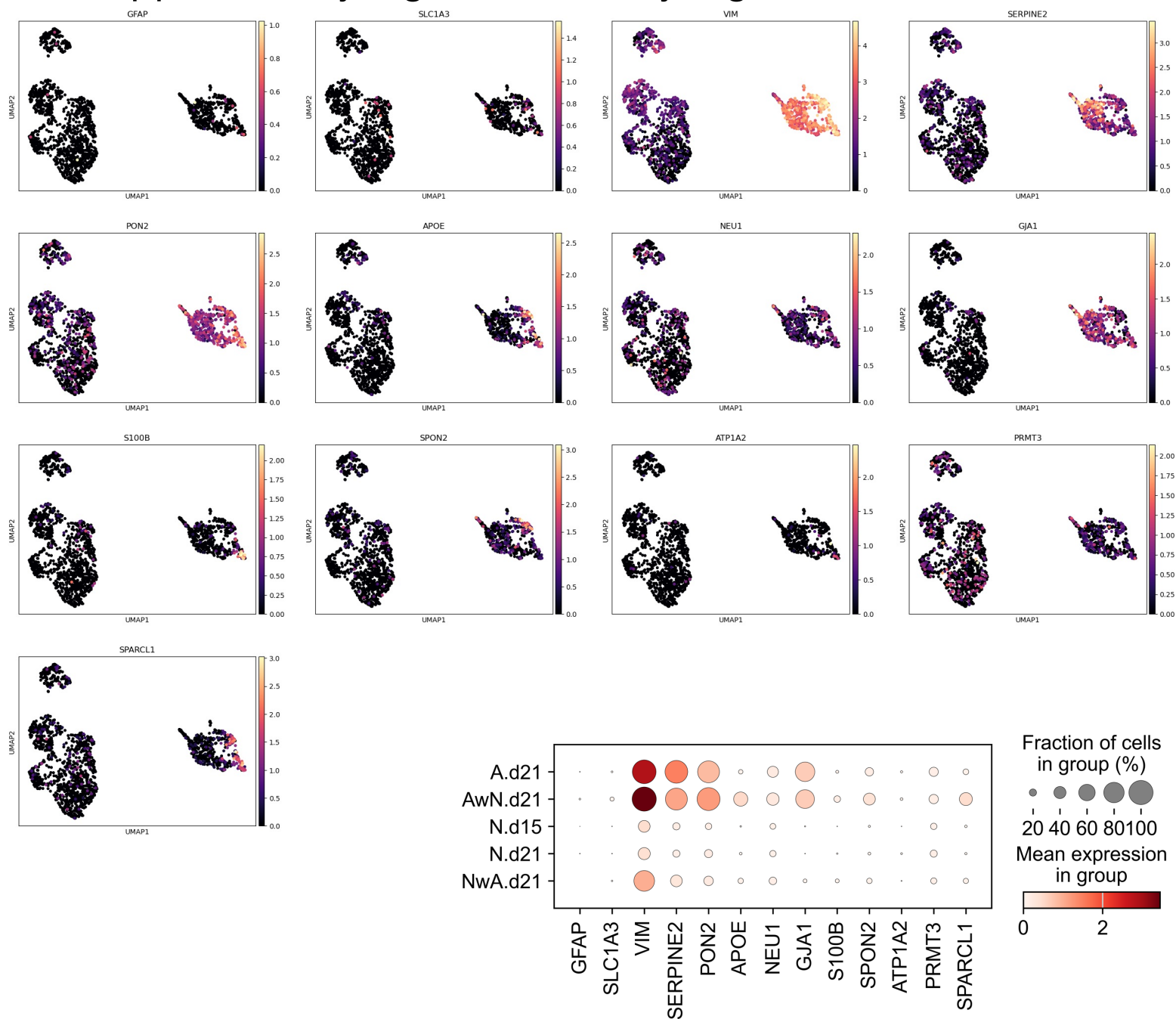

Supplement: Supplementary file 1 — Additional file 1: Sup. Figure 1. The fraction of the cells in each cell type and the mean expression of each gene in each cell type. [file 12915_2024_1867_MOESM1_ESM.pdf]

Supplementary Figure 2: Glutamatergic genes from table 1

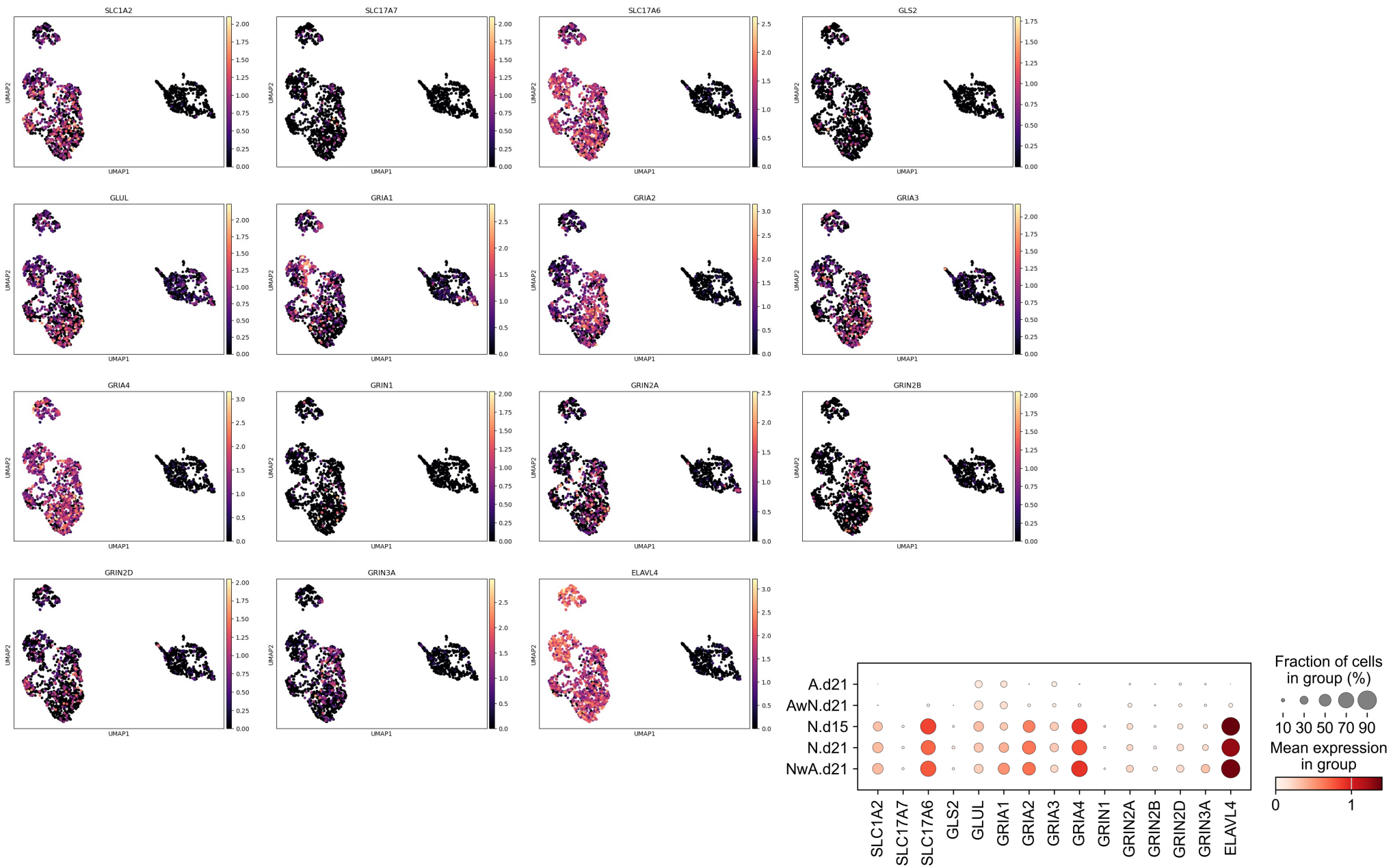

Supplement: Supplementary file 2 — Additional file 2: Sup. Figure 2. As above for glutamatergic genes. [file 12915_2024_1867_MOESM2_ESM.pdf]

# Supplementary Figure 3: Synaptic genes from table 1

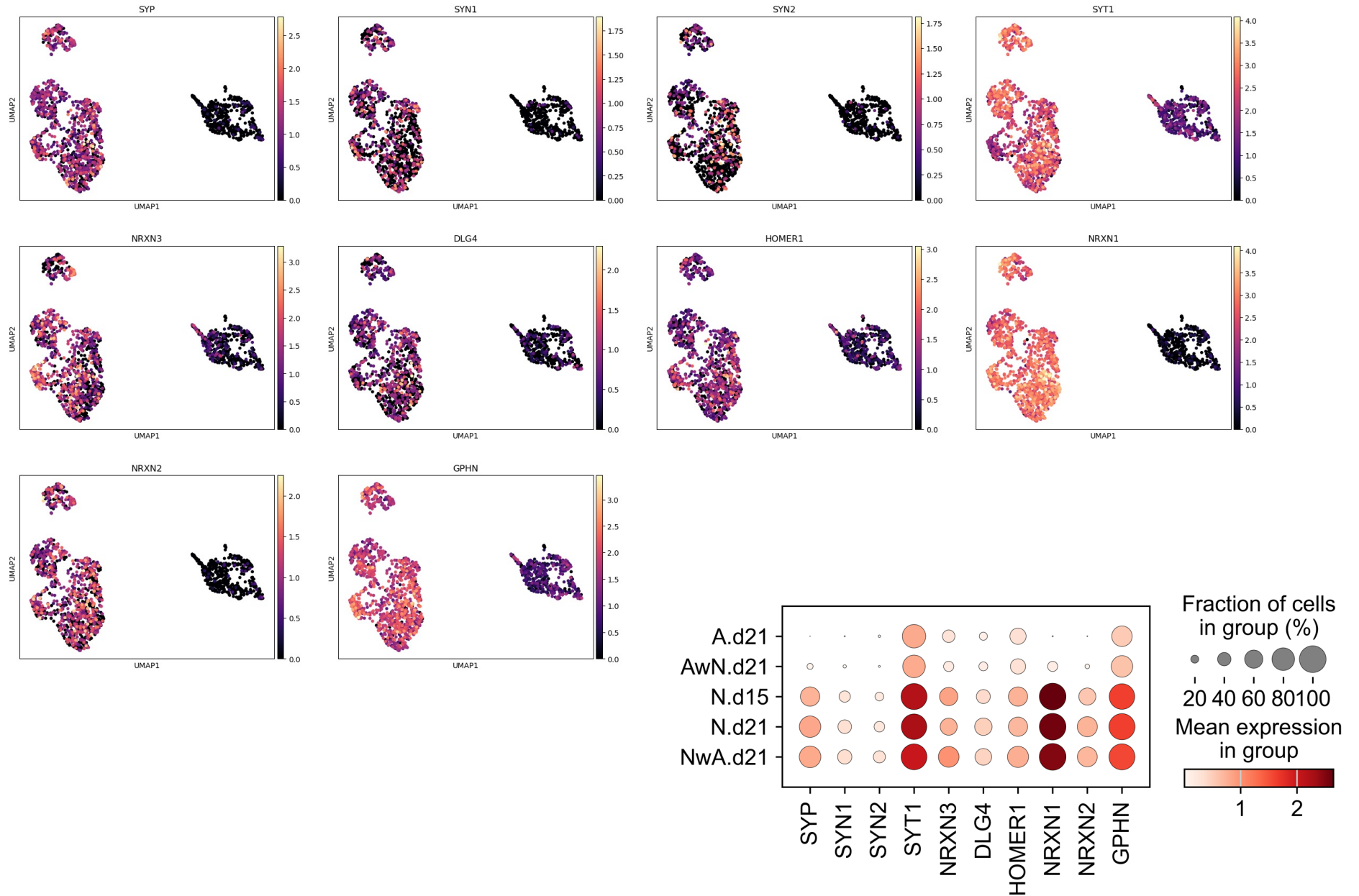

Supplement: Supplementary file 3 — Additional file 3: Sup. Figure 3. As above for synaptic genes. [file 12915_2024_1867_MOESM3_ESM.pdf]

# Supplementary Figure 4: Camk genes from table 1

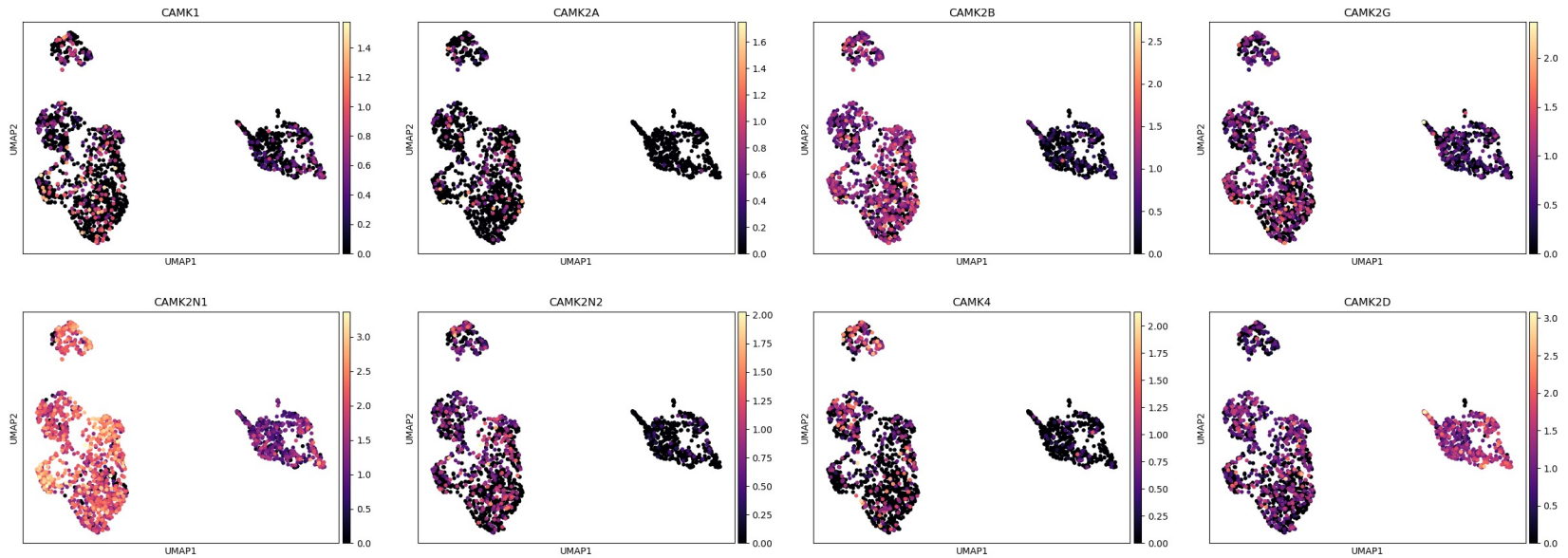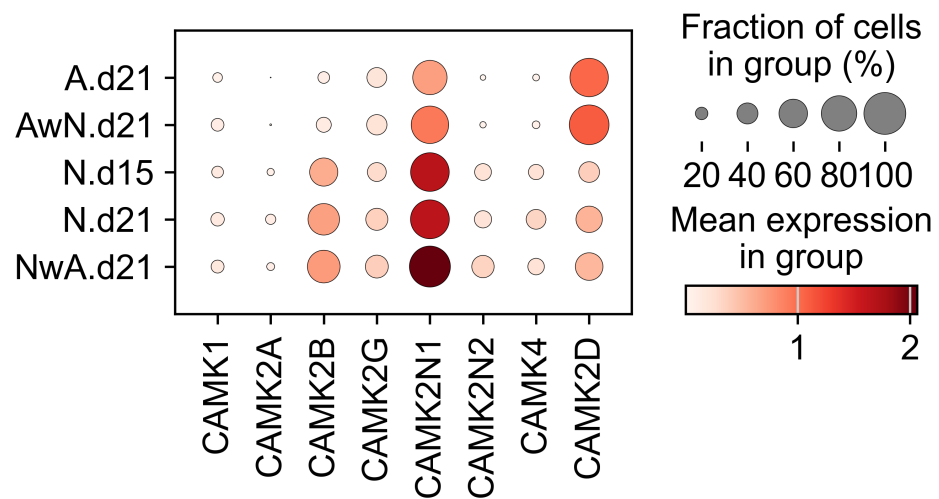

Supplement: Supplementary file 4 — Additional file 4: Sup. Figure 4. As above for CaM Kinase genes. [file 12915_2024_1867_MOESM4_ESM.pdf]

# Supplementary Figure 5: Other genes from table 1

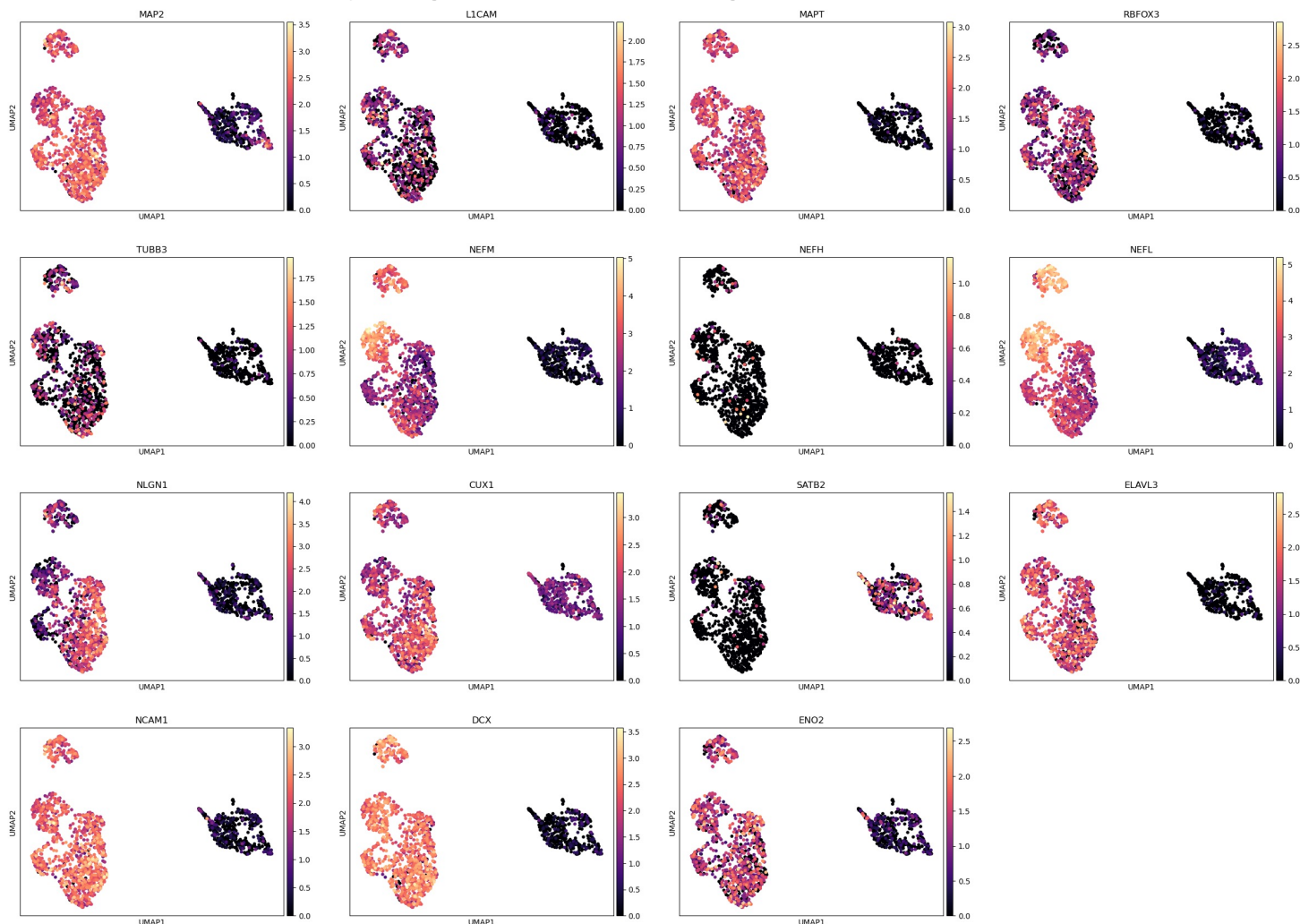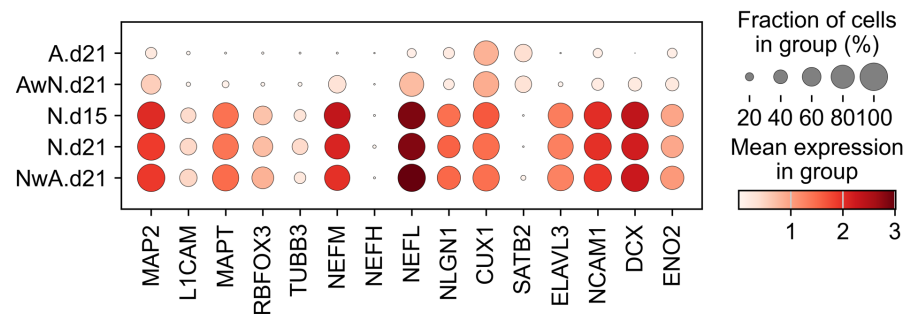

Supplement: Supplementary file 5 — Additional file 5: Sup. Figure 5. As above for other neuronal genes. [file 12915_2024_1867_MOESM5_ESM.pdf]

# Supplementary Figure 7

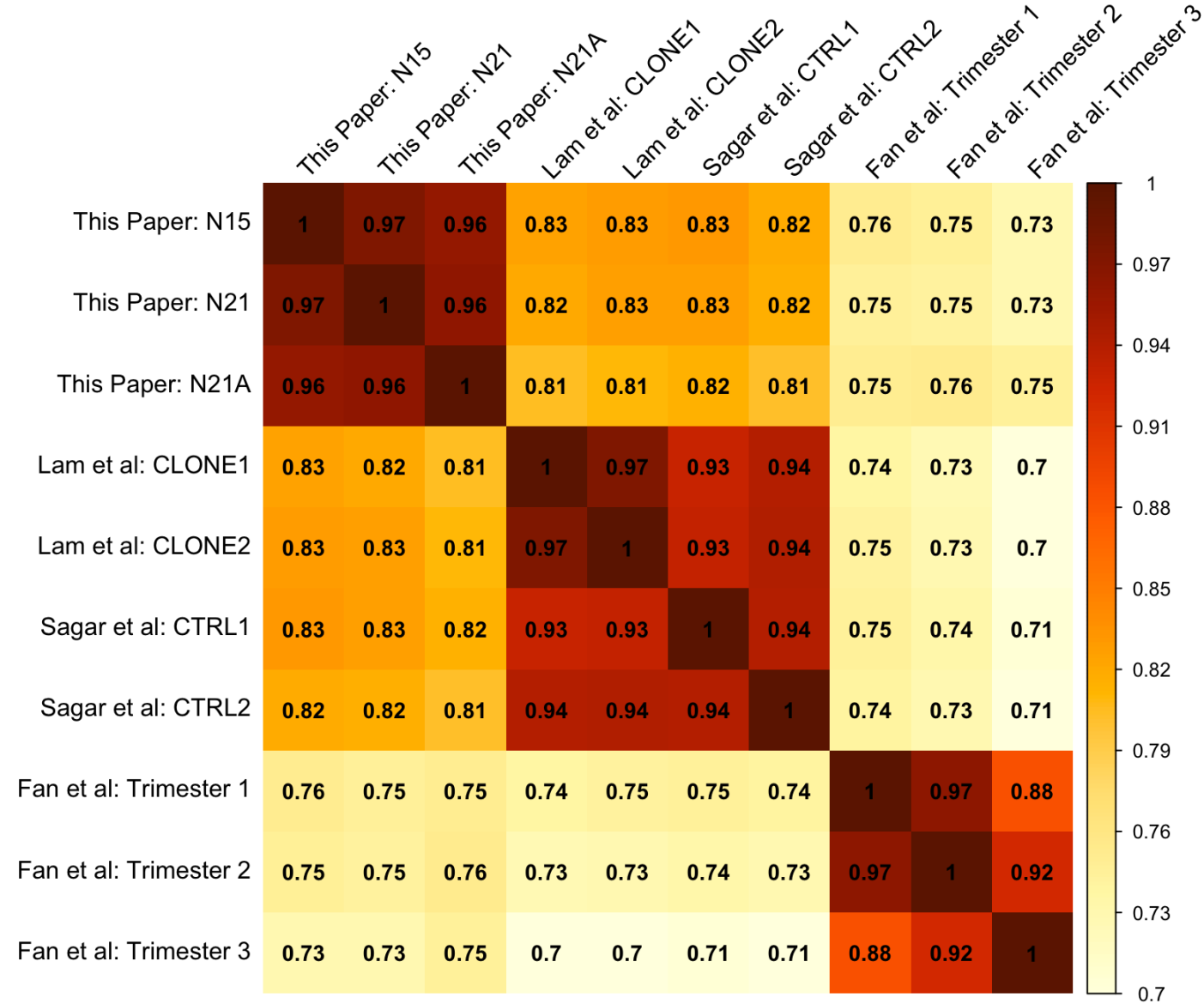

Spearman Correlation

Supplement: Supplementary file 6 — Additional file 6: Sup. Figure 6. Gene expression correlations with bulk dataset from other cell lines similarly differentiated in vitro to neurons and in one vivo dataset. [file 12915_2024_1867_MOESM6_ESM.pdf]

# Supplementary Figure 6

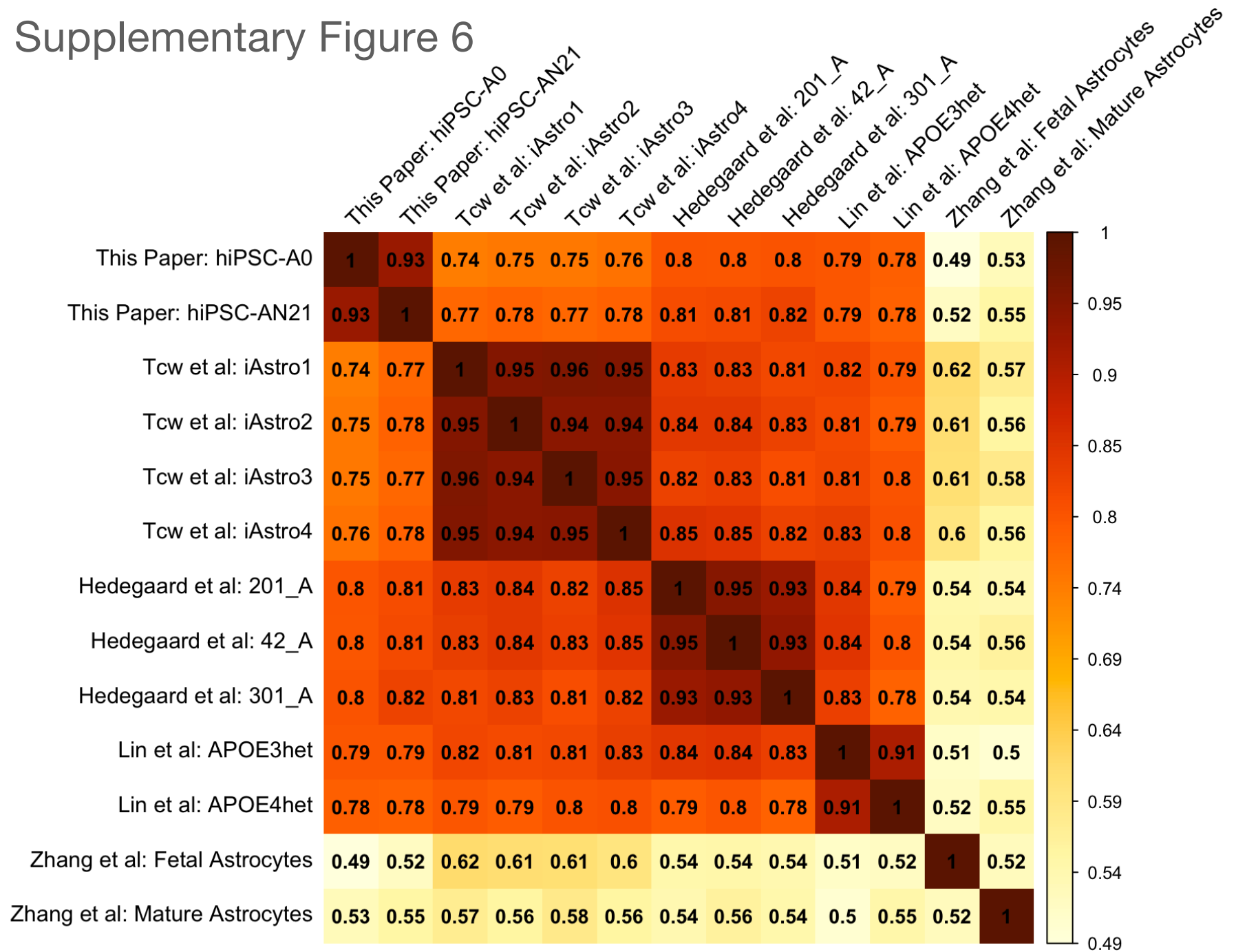

**Spearman  
Correlation**

Supplement: Supplementary file 7 — Additional file 7: Sup. Figure 7. Gene expression correlations with bulk dataset from other cell lines similarly differentiated in vitro to astrocytes and in one vivo dataset. [file 12915_2024_1867_MOESM7_ESM.pdf]

# GO term Enrichments – up in N15 vs N21

Terms

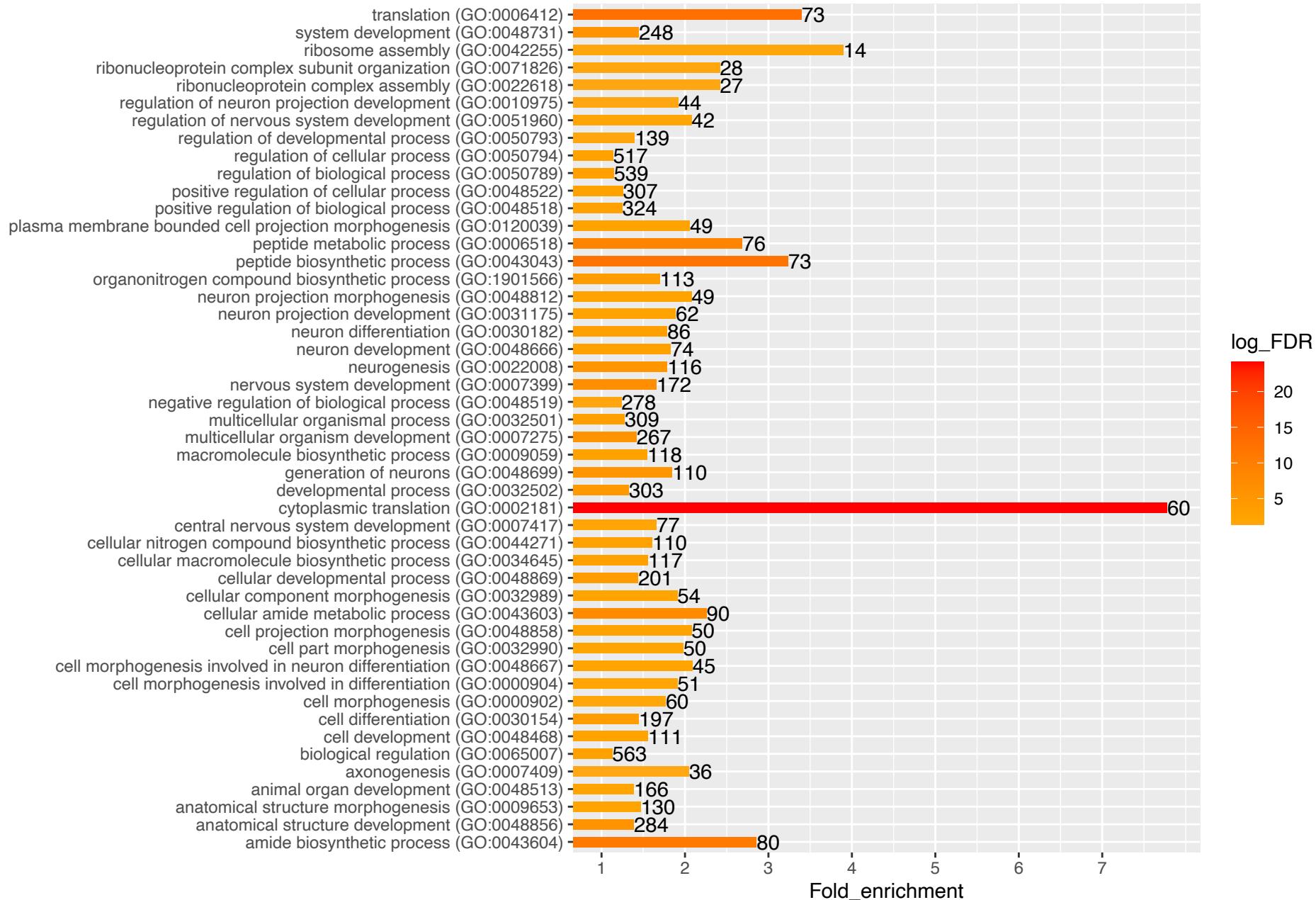

Supplement: Supplementary file 13 — Additional file 13: Sup. Figure 8. Supplementary Figure 8: PANTHER bioinformatics analysis of genes expressed higher in hiPSC-N15 than hiPSC-N21 shown as a bar graph. The length of each bar corresponds to fold-enrichment, the clor to statistical significance and the number next to it to the number of significantly differentially expressed genes in each pathway. [file 12915_2024_1867_MOESM13_ESM.pdf]

# GO term Enrichments – up in N21 vs N15

Terms

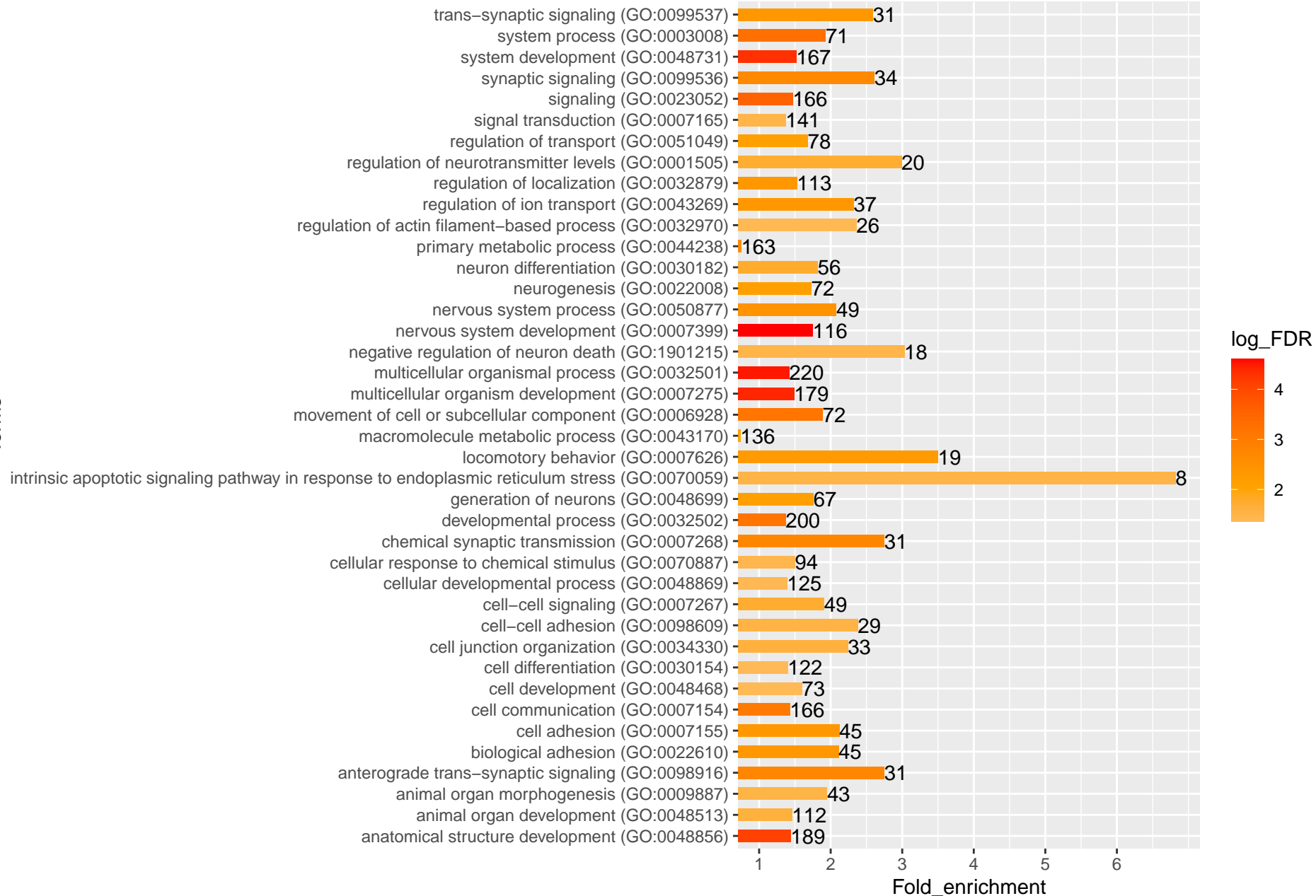

Supplement: Supplementary file 14 — Additional file 14: Sup. Figure 9. PANTHER bioinformatics analysis of genes expressed higher in hiPSC-N21 than hiPSC-N15 shown as in Supplementary Figure 8. [file 12915_2024_1867_MOESM14_ESM.pdf]

## GO term Enrichments – up in N21A vs N21

Terms

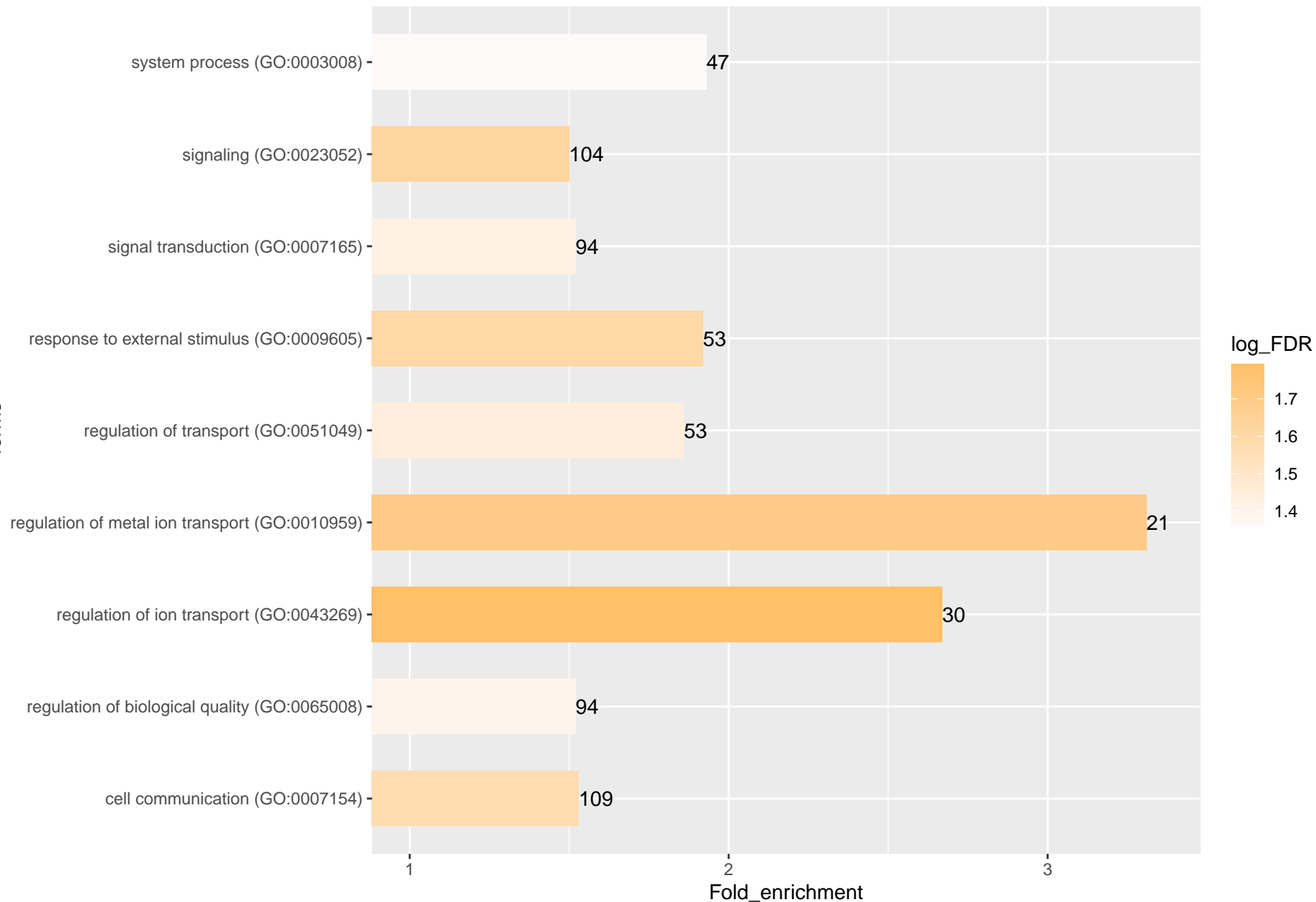

Supplement: Supplementary file 17 — Additional file 17: Sup. Figure 10. PANTHER bioinformatics analysis of genes expressed higher in hiPSC-N21A than hiPSC-N21 shown as in Supplementary Figure 8. [file 12915_2024_1867_MOESM17_ESM.pdf]

# GO term Enrichments – up in N21 vs N21A

Terms

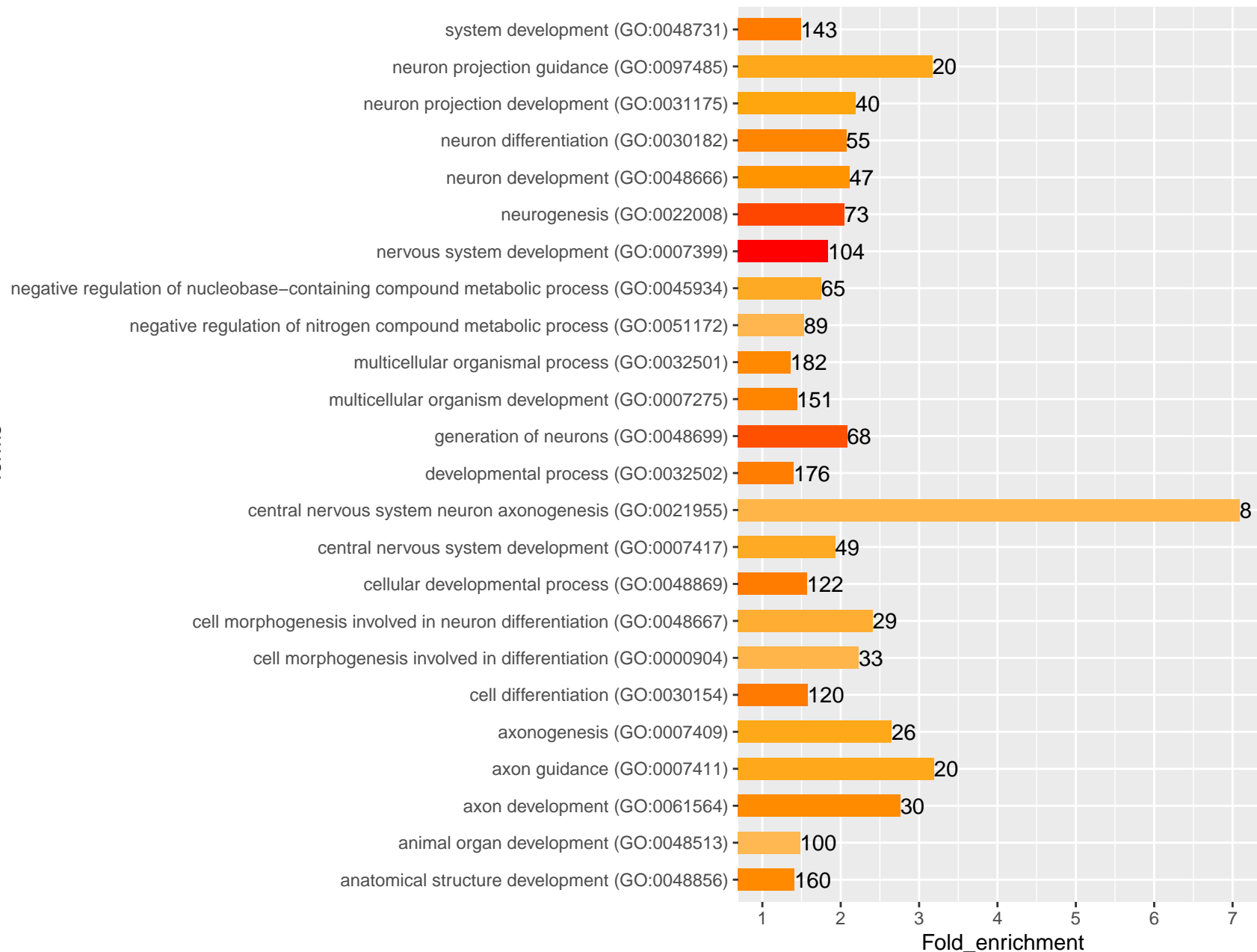

Supplement: Supplementary file 18 — Additional file 18: Sup. Figure 11. PANTHER bioinformatics analysis of genes expressed higher in hiPSC-N21 than hiPSC-N21A shown as in Supplementary Figure 8. [file 12915_2024_1867_MOESM18_ESM.pdf]

# GO term Enrichments – up in AN21 vs A0

Terms

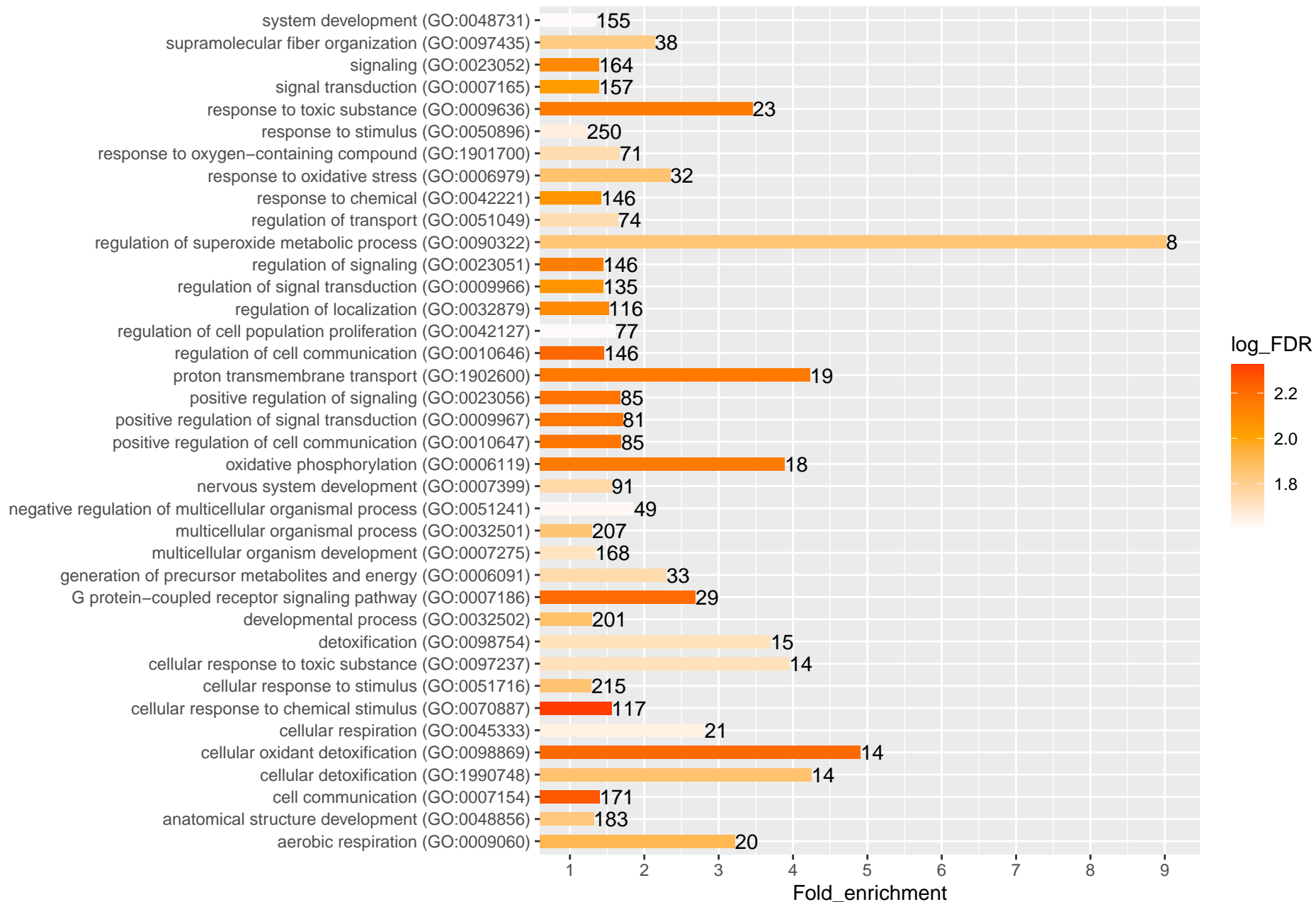

Supplement: Supplementary file 19 — Additional file 19: SuppTable8. Differential expression analysis between hiPSC-A0 and hiPSC-AN21. [file 12915_2024_1867_MOESM19_ESM.pdf]

# GO term Enrichments – up in A0 vs AN21

Terms

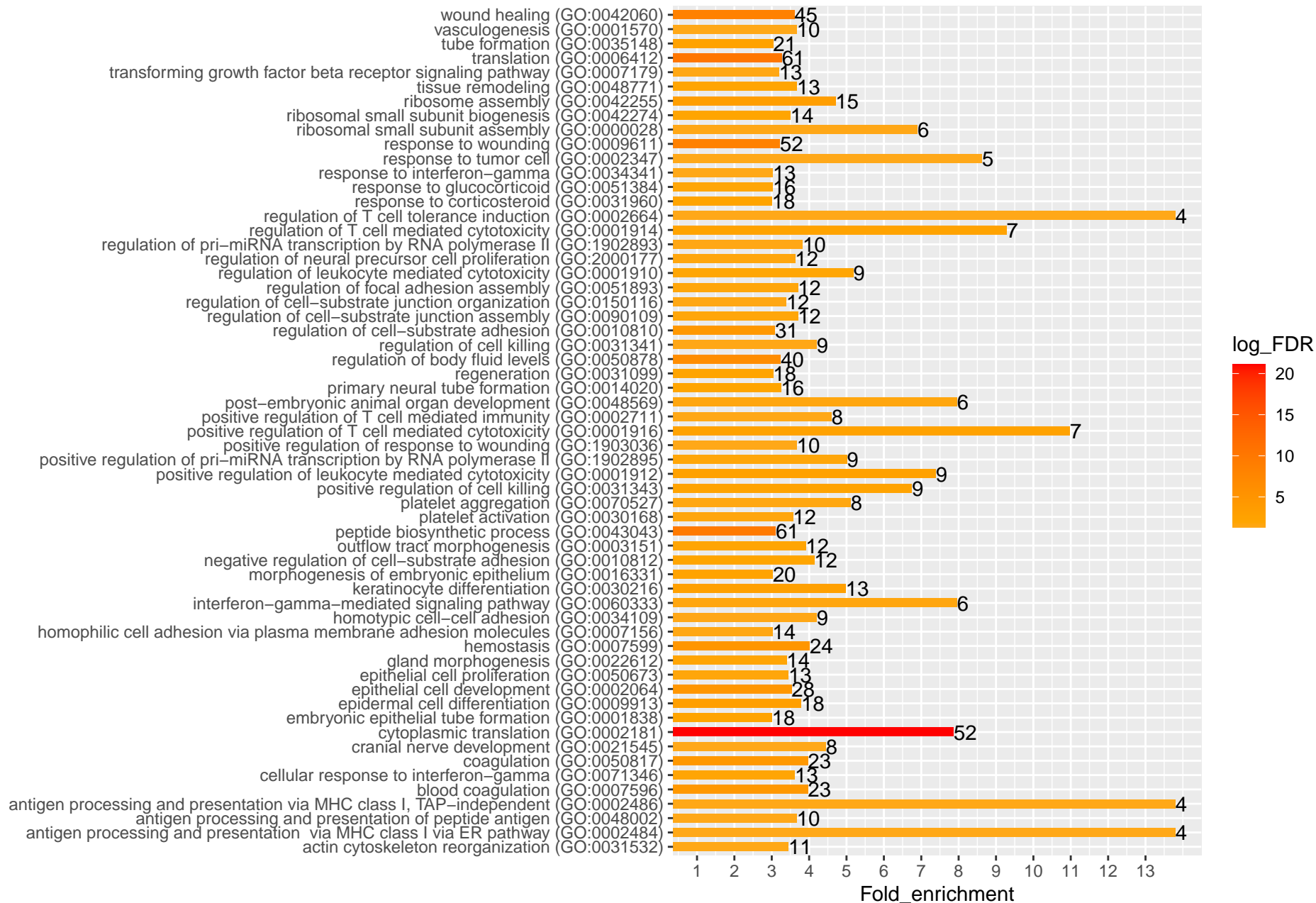

Supplement: Supplementary file 22 — Additional file 22: Sup. Figure 13. PANTHER bioinformatics analysis of genes expressed significantly higher in hiPSC-A0 than hiPSC-AN21 shown as in Supplementary Figure 8. Only more than 3-fold enrichments are shown. All enrichments are in sip. Table 9. [file 12915_2024_1867_MOESM22_ESM.pdf]

Supplementary Figure 15

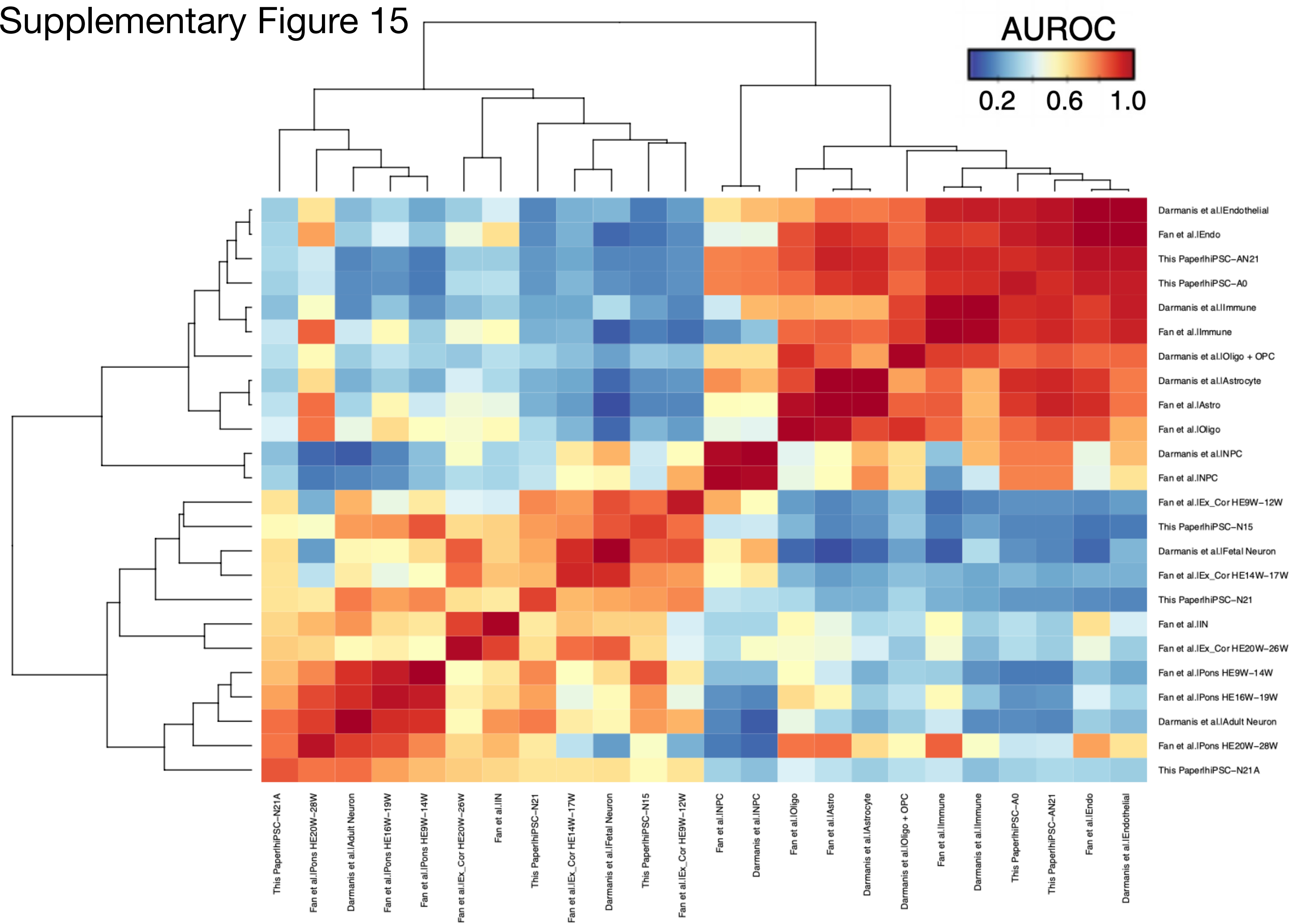

Supplement: Supplementary file 24 — Additional file 24: Sup. Figure 15. The complete heatmap of the MetaNeighbor analysis shown in Figure 2. MetaNeighbor analysis of our bulk and pseudobulk expression data with in vivo data from two in vivo studies. Ex_Cor, excitatory cortical; HEW, Human embryo week; Astro, astrocytes; Oligo, oligodendrocytes; OPC oligodendrocyte precursor cells; Endo, endothelial. [file 12915_2024_1867_MOESM24_ESM.pdf]

**A**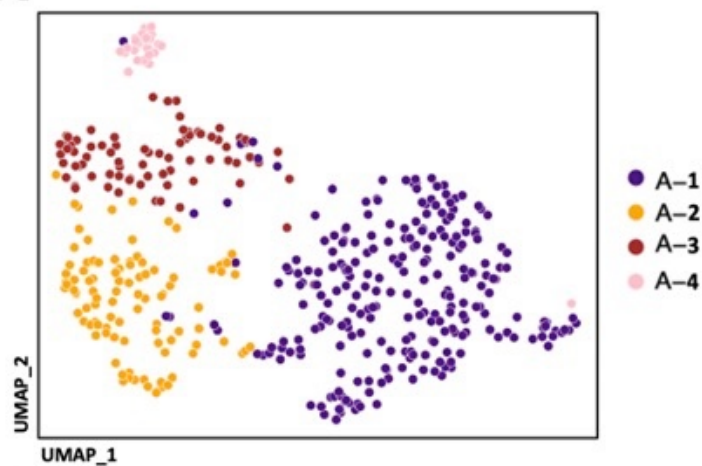**B**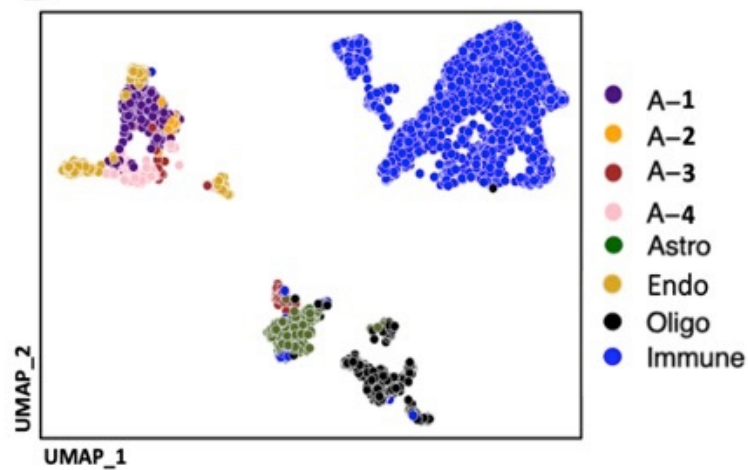**C**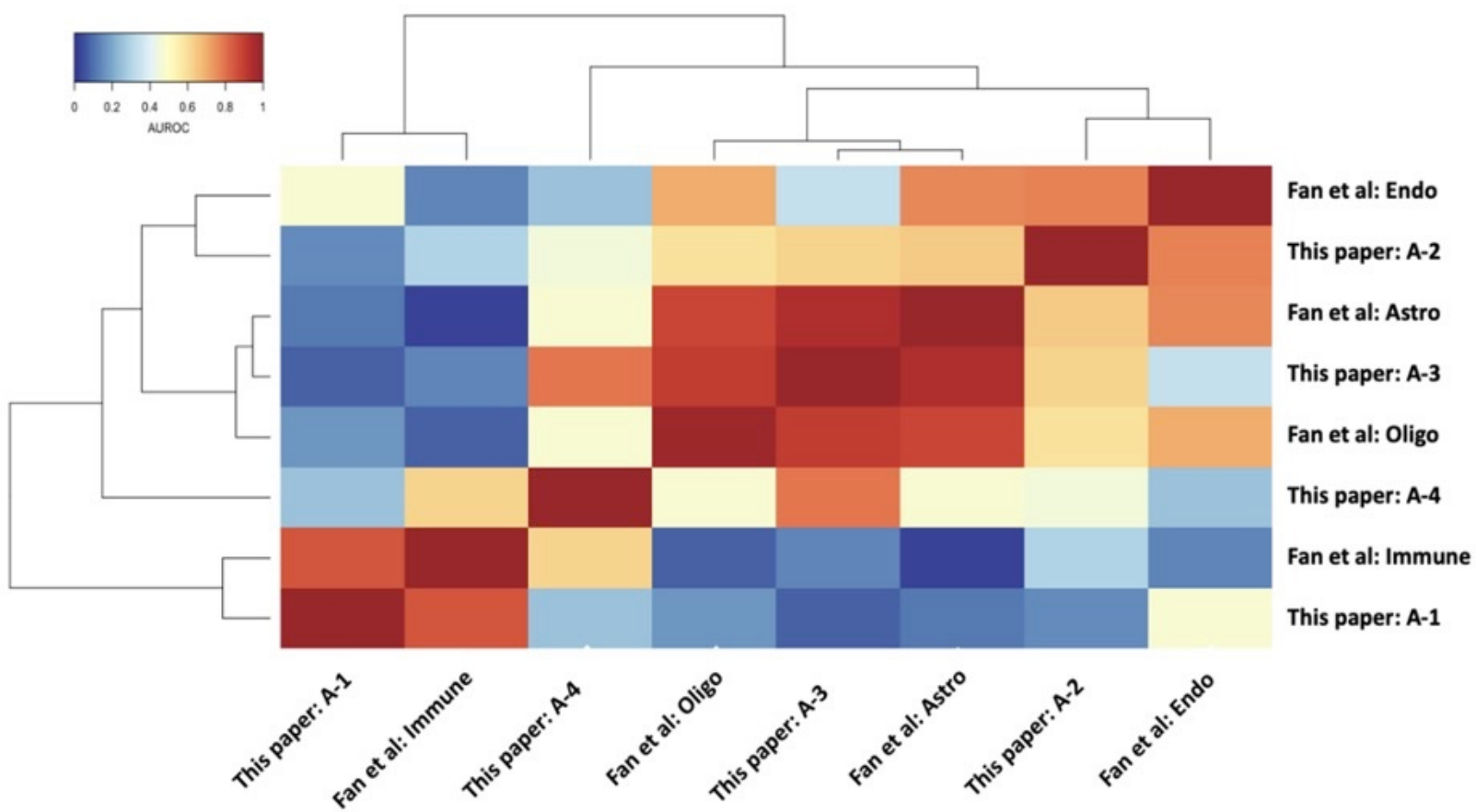

Supplement: Supplementary file 25 — Additional file 25: Sup. Figure 16. hiPSC-A cells re-clustered alone (A). Integrated UMAP (B) and MetaNeighbor analysis with Fan et al. show more similarity to in vivo astrocytes. [file 12915_2024_1867_MOESM25_ESM.pdf]

**A**

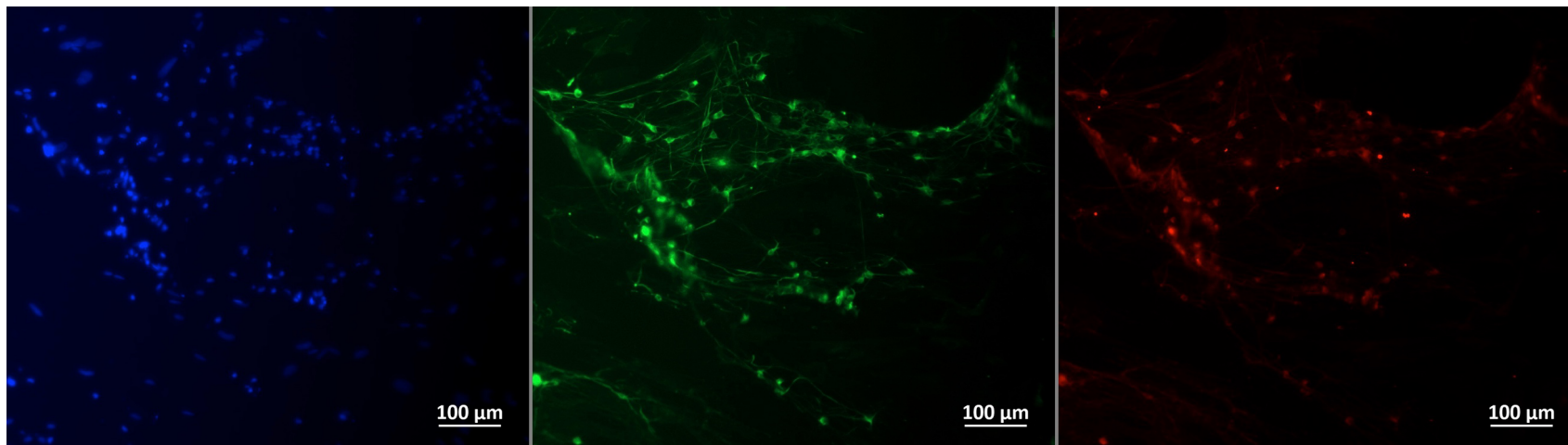

**B**

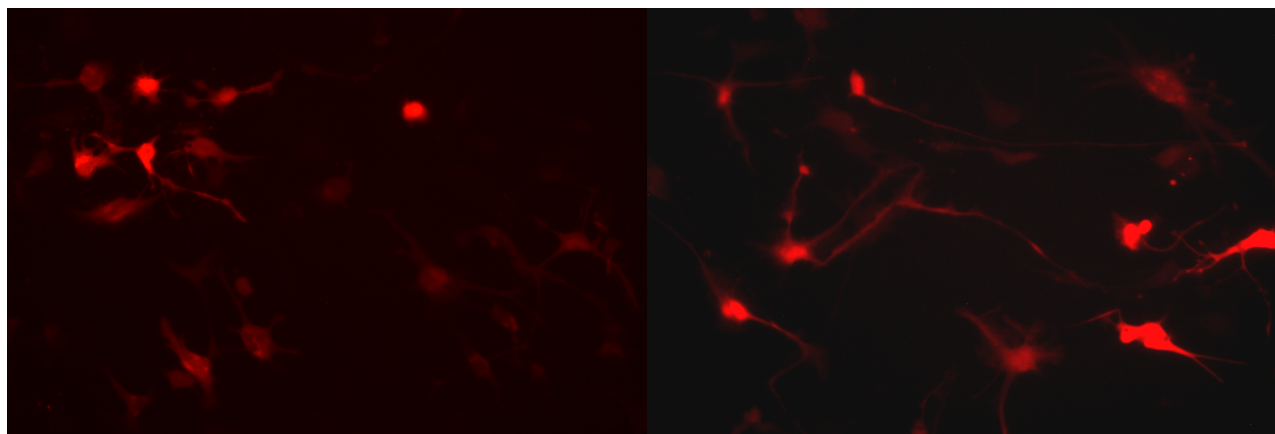

**C**

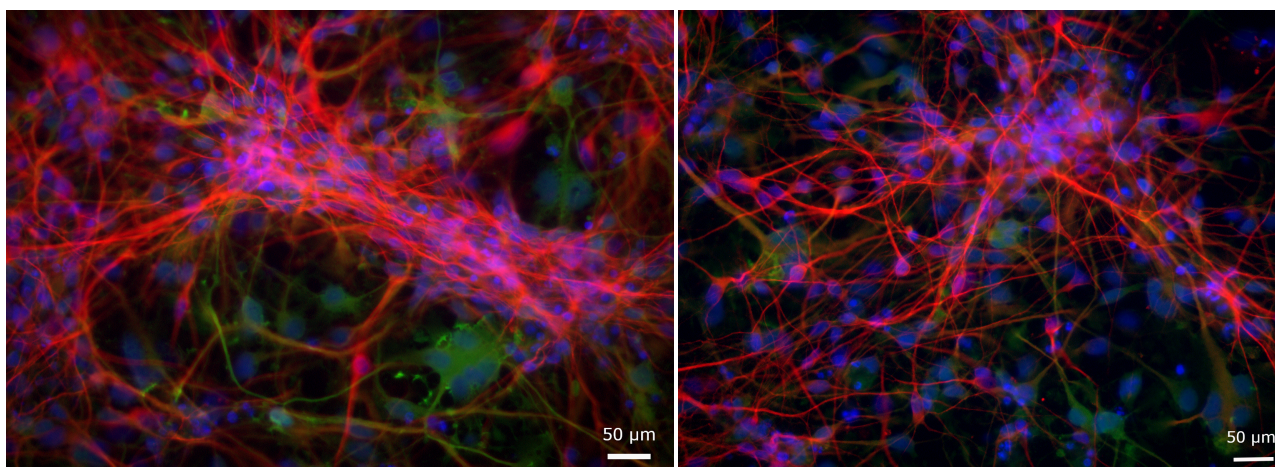

Supplement: Supplementary file 26 — Additional file 26: Sup. Figure 17. Staining of BC1 cells differentiated into neurons and astrocyte using the same protocols described here. A. Neurons growing in isolation. blue stain = DAPI, green stain = MAP2, Red stain = NeuN. B. Culture of induced astrocytes. Red stain = GFAP. Scale in B is as in C. C. Induced neurons growing in co-culture with induced astrocytes. Red stain = MAP2, green stain = GFAP, blue stain = DAPI. More images of neurons can be in our previously published work (references are in the text). [file 12915_2024_1867_MOESM26_ESM.pdf]
