# Supplementary material for: Insights for disease modeling from single-cell transcriptomics of iPSC-derived Ngn2-induced neurons and astrocytes across differentiation time and co-culture
Source: BMC Biol. 2024 Apr 2;22:75. doi: 10.1186/s12915-024-01867-4 (PMC10985965; doi:10.1186/s12915-024-01867-4)
Supplement: Supplementary file 9 — Additional file 9: SuppTable2. Graphical representation of PANTHER bioinformatics analysis of genes showing differential expression between neuronal clusters from Table 1. All comparisons are shown together in columns. Colored cells indicate significant enrichment for that comparison and the color indicates whether it is for up-regulated genes *red), Downregulated genes (blue) or both up and down regulated genes (yellow). [file 12915_2024_1867_MOESM9_ESM.pdf]

		N-I	N-II	N-III	N-IV	N-V
cell-cell adhesion	(GO:0098609)					
nervous system development	(GO:0007399)					
anatomical structure morphogenesis	(GO:0009653)					
neurogenesis	(GO:0022008)					
cell differentiation	(GO:0030154)					
neuron differentiation	(GO:0030182)					
cell development	(GO:0048468)					
generation of neurons	(GO:0048699)					
system development	(GO:0048731)					
cellular developmental process	(GO:0048869)					
synaptic signaling	(GO:0099536)					
trans-synaptic signaling	(GO:0099537)					
multicellular organism development	(GO:0007275)					
multicellular organismal process	(GO:0032501)					
developmental process	(GO:0032502)					
chemical synaptic transmission	(GO:0007268)					
anterograde trans-synaptic signaling	(GO:0098916)					
cell morphogenesis	(GO:0000902)					
neuron projection development	(GO:0031175)					
cellular component morphogenesis	(GO:0032989)					
cell part morphogenesis	(GO:0032990)					
neuron development	(GO:0048666)					
neuron projection morphogenesis	(GO:0048812)					
cell projection morphogenesis	(GO:0048858)					
plasma membrane bounded cell projection morphogenesis	(GO:0120039)					
anatomical structure development	(GO:0048856)					
cell morphogenesis involved in differentiation	(GO:0000904)					
chemotaxis	(GO:0006935)					
axonogenesis	(GO:0007409)					
axon guidance	(GO:0007411)					
locomotion	(GO:0040011)					
taxis	(GO:0042330)					
cell morphogenesis involved in neuron differentiation	(GO:0048667)					
axon development	(GO:0061564)					
neuron projection guidance	(GO:0097485)					
modulation of chemical synaptic transmission	(GO:0050804)					
regulation of trans-synaptic signaling	(GO:0099177)					
cell-cell adhesion via plasma-membrane adhesion molecules	(GO:0098742)					
regulation of cellular process	(GO:0050794)					
cell adhesion	(GO:0007155)					
regulation of neuron projection development	(GO:0010975)					
response to chemical	(GO:0042221)					
regulation of axonogenesis	(GO:0050770)					
cell-cell signaling	(GO:0007267)					
response to external stimulus	(GO:0009605)					
cell projection organization	(GO:0030030)					
biological regulation	(GO:0065007)					
plasma membrane bounded cell projection organization	(GO:0120036)					
cell communication	(GO:0007154)					
signaling	(GO:0023052)					

cellular process	(GO:0009987)					
cellular component organization or biogenesis	(GO:0071840)					
cell junction organization	(GO:0034330)					
translation	(GO:0006412)					
peptide metabolic process	(GO:0006518)					
peptide biosynthetic process	(GO:0043043)					
amide biosynthetic process	(GO:0043604)					
regulation of sodium ion transport	(GO:0002028)					
biological_process	(GO:0008150)					
ribonucleoprotein complex biogenesis	(GO:0022613)					
ribosome biogenesis	(GO:0042254)					
vesicle-mediated transport in synapse	(GO:0099003)					
synaptic vesicle cycle	(GO:0099504)					
synaptic transmission, glutamatergic	(GO:0035249)					
central nervous system development	(GO:0007417)					
protein-RNA complex assembly	(GO:0022618)					
regulation of RNA splicing	(GO:0043484)					
regulation of mRNA splicing, via spliceosome	(GO:0048024)					
regulation of mRNA processing	(GO:0050684)					
maintenance of location in cell	(GO:0051651)					
adherens junction organization	(GO:0034332)					
regulation of transport	(GO:0051049)					
cytoplasmic translation	(GO:0002181)					
regulation of anatomical structure morphogenesis	(GO:0022603)					
monoatomic ion transmembrane transport	(GO:0034220)					
synaptic vesicle recycling	(GO:0036465)					
ribosomal large subunit biogenesis	(GO:0042273)					
ribosomal small subunit biogenesis	(GO:0042274)					
regulation of cell differentiation	(GO:0045595)					
synaptic vesicle endocytosis	(GO:0048488)					
animal organ development	(GO:0048513)					
regulation of neurogenesis	(GO:0050767)					
regulation of developmental process	(GO:0050793)					
regulation of nervous system development	(GO:0051960)					
regulation of biological quality	(GO:0065008)					
protein-RNA complex organization	(GO:0071826)					
regulation of biological process	(GO:0050789)					
calcium-ion regulated exocytosis	(GO:0017156)					
regulation of cell migration	(GO:0030334)					
cell junction assembly	(GO:0034329)					
heterophilic cell-cell adhesion via plasma membrane cell adhesion molecules	(GO:0007157)					
regulation of cell projection organization	(GO:0031344)					
receptor clustering	(GO:0043113)					
response to stimulus	(GO:0050896)					
regulation of plasma membrane bounded cell projection organization	(GO:0120035)					
signal transduction	(GO:0007165)					
biosynthetic process	(GO:0009058)					
macromolecule biosynthetic process	(GO:0009059)					
cellular biosynthetic process	(GO:0044249)					
cellular nitrogen compound biosynthetic process	(GO:0044271)					
synapse organization	(GO:0050808)					

organonitrogen compound biosynthetic process	(GO:1901566)					
organic substance biosynthetic process	(GO:1901576)					
regulation of metal ion transport	(GO:0010959)					
neurotransmitter transport	(GO:0006836)					
response to oxidative stress	(GO:0006979)					
neurotransmitter secretion	(GO:0007269)					
gene expression	(GO:0010467)					
synaptic vesicle exocytosis	(GO:0016079)					
signal release	(GO:0023061)					
mitochondrial respiratory chain complex I assembly	(GO:0032981)					
amide metabolic process	(GO:0043603)					
regulated exocytosis	(GO:0045055)					
signal release from synapse	(GO:0099643)					
carbohydrate derivative biosynthetic process	(GO:1901137)					
organonitrogen compound metabolic process	(GO:1901564)					
negative regulation of protein phosphorylation	(GO:0001933)					
cellular component organization	(GO:0016043)					
negative regulation of phosphorylation	(GO:0042326)					
negative regulation of phosphorus metabolic process	(GO:0010563)					
negative regulation of protein modification process	(GO:0031400)					
negative regulation of phosphate metabolic process	(GO:0045936)					
regulation of alternative mRNA splicing, via spliceosome	(GO:0000381)					
translational elongation	(GO:0006414)					
regulation of exocytosis	(GO:0017157)					
regulation of localization	(GO:0032879)					
regulation of transmembrane transport	(GO:0034762)					
regulation of monoatomic ion transmembrane transport	(GO:0034765)					
regulation of monoatomic ion transport	(GO:0043269)					
maintenance of location	(GO:0051235)					
import into cell	(GO:0098657)					
regulation of monoatomic cation transmembrane transport	(GO:1904062)					
regulation of sodium ion transmembrane transporter activity	(GO:2000649)					
cell-cell junction assembly	(GO:0007043)					
regulation of cell growth	(GO:0001558)					
intermediate filament-based process	(GO:0045103)					
intermediate filament cytoskeleton organization	(GO:0045104)					
intermediate filament organization	(GO:0045109)					
intermediate filament bundle assembly	(GO:0045110)					
