# Supplementary material for: Insights for disease modeling from single-cell transcriptomics of iPSC-derived Ngn2-induced neurons and astrocytes across differentiation time and co-culture
Source: BMC Biol. 2024 Apr 2;22:75. doi: 10.1186/s12915-024-01867-4 (PMC10985965; doi:10.1186/s12915-024-01867-4)
Supplement: Supplementary file 10 — Additional file 10: SuppTable3. Graphical representation of PANTHER bioinformatics analysis of genes showing differential expression between astrocytic clusters from Table 1. These are shown as in Supplementary Table 2. Note that there were no enrichments for up-regulated genes, which is why ll cells are blue. [file 12915_2024_1867_MOESM10_ESM.pdf]

		A-I	A-II	A-III
anatomical structure development	(GO:0048856)	■		■
anatomical structure morphogenesis	(GO:0009653)	■		■
biological_process	(GO:0008150)	■		■
cell adhesion	(GO:0007155)	■		■
cell differentiation	(GO:0030154)	■		■
cell morphogenesis involved in differentiation	(GO:0000904)	■		■
cellular developmental process	(GO:0048869)	■		■
cellular process	(GO:0009987)	■		■
developmental process	(GO:0032502)	■		■
external encapsulating structure organization	(GO:0045229)	■		■
extracellular matrix organization	(GO:0030198)	■		■
extracellular structure organization	(GO:0043062)	■		■
multicellular organism development	(GO:0007275)	■		■
multicellular organismal process	(GO:0032501)	■		■
neuron development	(GO:0048666)	■		■
neuron differentiation	(GO:0030182)	■		■
regulation of cell communication	(GO:0010646)	■		■
regulation of signal transduction	(GO:0009966)	■		■
regulation of signaling	(GO:0023051)	■		■
system development	(GO:0048731)	■		■
aerobic electron transport chain	(GO:0019646)	■		
aerobic respiration	(GO:0009060)	■		
ATP biosynthetic process	(GO:0006754)	■		
cell-cell adhesion	(GO:0098609)	■		
cellular respiration	(GO:0045333)	■		
cytoplasmic translation	(GO:0002181)	■		
generation of precursor metabolites and energy	(GO:0006091)	■		
organonitrogen compound biosynthetic process	(GO:1901566)	■		
oxidative phosphorylation	(GO:0006119)	■		
proton motive force-driven ATP synthesis	(GO:0015986)	■		
regulation of intracellular signal transduction	(GO:1902531)	■		
response to chemical	(GO:0042221)	■		
response to endogenous stimulus	(GO:0009719)	■		
actin cytoskeleton organization	(GO:0030036)			■
actin filament organization	(GO:0007015)			■
actin filament-based process	(GO:0030029)			■
cell development	(GO:0048468)			■
cell morphogenesis	(GO:0000902)			■
generation of neurons	(GO:0048699)			■
nervous system development	(GO:0007399)			■
neurogenesis	(GO:0022008)			■
neuron projection development	(GO:0031175)			■
regulation of developmental process	(GO:0050793)			■
supramolecular fiber organization	(GO:0097435)			■
