# Supplementary material for: Insights for disease modeling from single-cell transcriptomics of iPSC-derived Ngn2-induced neurons and astrocytes across differentiation time and co-culture
Source: BMC Biol. 2024 Apr 2;22:75. doi: 10.1186/s12915-024-01867-4 (PMC10985965; doi:10.1186/s12915-024-01867-4)
Supplement: Supplementary file 23 — Additional file 23: Sup. Figure 14. The top 50 genes in each cell type contrast, detailed in the ““Differences between hiPSC-N15 and hiPSC-N21,” “Differences between hiPSC-N21 and hiPSC-N21A,” and “Differences between hiPSC-A0 and hiPSC-AN21” sections shown in heatmaps. [file 12915_2024_1867_MOESM23_ESM.pdf]

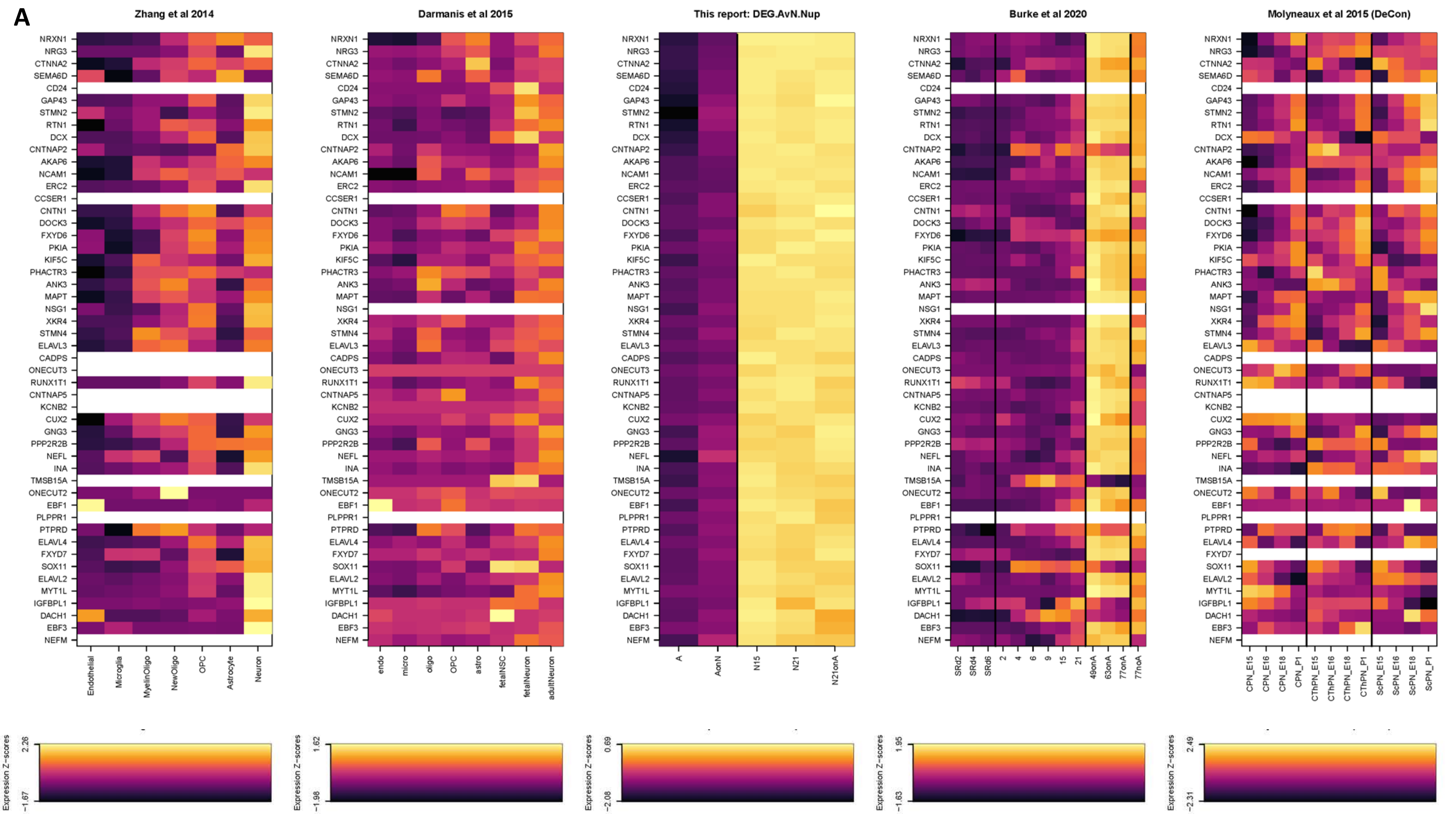
A
Zhang et al 2014
Darmanis et al 2015
This report: DEG.AvN.Nup
Burke et al 2020
Molyneaux et al 2015 (DeCon)
NRXN1
NRG3
CTNNA2
SEMA6D
CD24
GAP43
STMN2
RTN1
DCX
CNTNAP2
AKAP6
NCAM1
ERC2
CCSER1
CNTN1
DOCK3
FXYD6
PKIA
KIF5C
PHACTR3
ANK3
MAPT
NSG1
XKR4
STMN4
ELAVL3
CADPS
ONECUT3
RUNX1T1
CNTNAP5
KCNB2
CUX2
GNG3
PPP2R2B
NEFL
INA
TMSB15A
ONECUT2
EBF1
PLPPR1
PTPRD
ELAVL4
FXYD7
SOX11
ELAVL2
MYT1L
IGFBPL1
DACH1
EBF3
NEFM
Endothelial
Microglia
MyelinOligo
NewOligo
OPC
Astrocyte
Neuron
endo
micro
oligo
OPC
astro
fetalNSC
fetalNeuron
adultNeuron
A
AonN
N15
N21
N21onA
SRd2
SRd4
SRd6
2
4
6
9
15
21
49onA
63onA
77onA
77noA
CPN_E15
CPN_E16
CPN_E18
CPN_P1
CThPN_E15
CThPN_E16
CThPN_E18
CThPN_P1
ScPN_E15
ScPN_E16
ScPN_E18
ScPN_P1
Expression Z-scores
2.26
-1.67
1.62
-1.98
0.69
-2.08
1.95
-1.63
2.49
-2.31

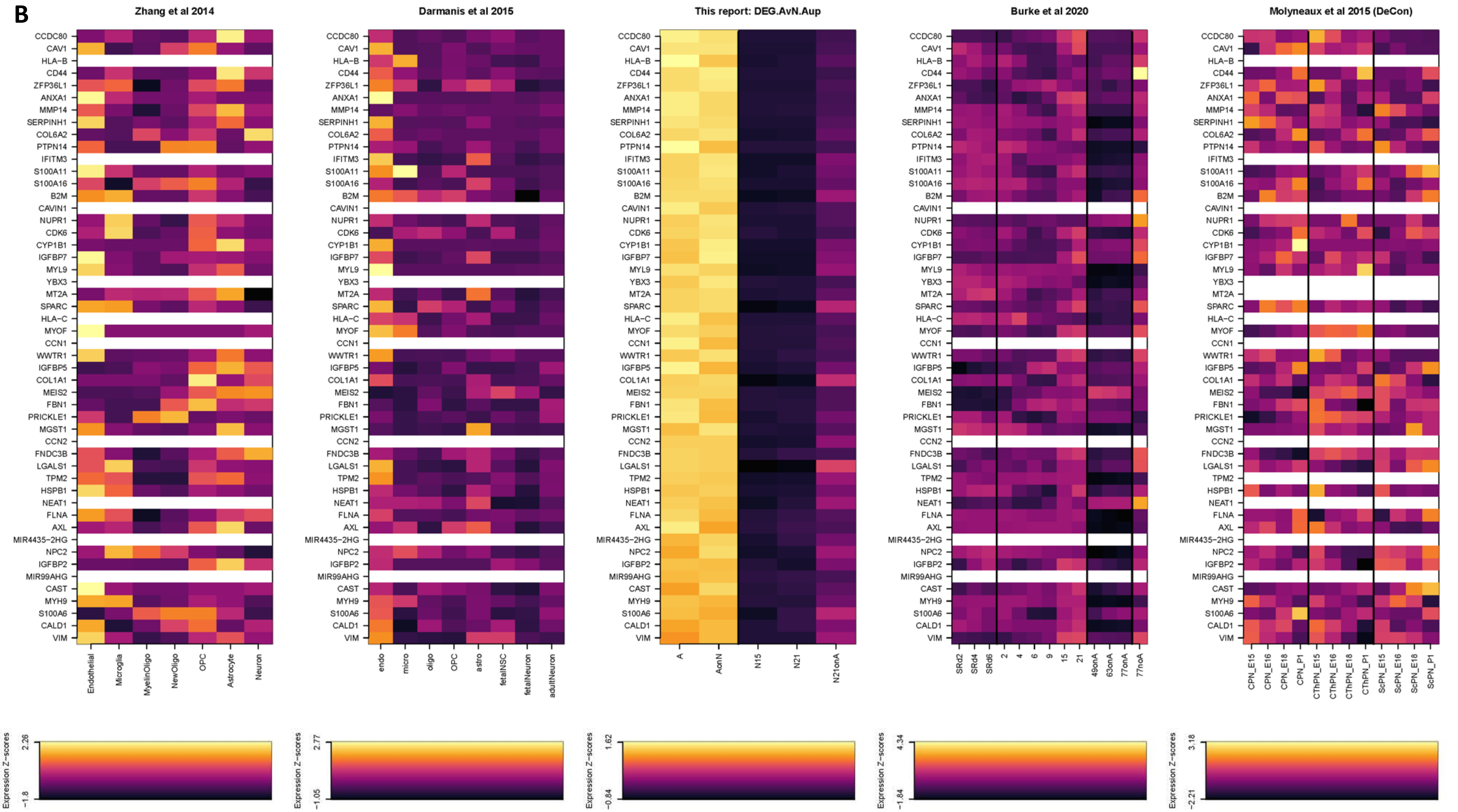
B
Zhang et al 2014
Darmanis et al 2015
This report: DEG.AvN.Aup
Burke et al 2020
Molyneaux et al 2015 (DeCon)
CCDC80
CAV1
HLA-B
CD44
ZFP36L1
ANXA1
MMP14
SERPINH1
COL6A2
PTPN14
IFITM3
S100A11
S100A16
B2M
CAVIN1
NUPR1
CDK6
CYP1B1
IGFBP7
MYL9
YBX3
MT2A
SPARC
HLA-C
MYOF
CCN1
WWTR1
IGFBP5
COL1A1
MEIS2
FBN1
PRICKLE1
MGST1
CCN2
FNDC3B
LGALS1
TPM2
HSPB1
NEAT1
FLNA
AXL
MIR4435-2HG
NPC2
IGFBP2
MIR99AHG
CAST
MYH9
S100A6
CALD1
VIM
Endothelial
Microglia
MyelinOligo
NewOligo
OPC
Astrocyte
Neuron
endo
micro
oligo
OPC
astro
fetalNSC
fetalNeuron
adultNeuron
A
AonN
N15
N21
N21onA
SRd2
SRd4
SRd6
2
4
6
9
15
21
49onA
63onA
77onA
77noA
CPN_E15
CPN_E16
CPN_E18
CPN_P1
CThPN_E15
CThPN_E16
CThPN_E18
CThPN_P1
ScPN_E15
ScPN_E16
ScPN_E18
ScPN_P1
Expression Z-scores
2.26
-1.8
Expression Z-scores
2.77
-1.05
Expression Z-scores
1.62
-0.84
Expression Z-scores
4.34
-1.84
Expression Z-scores
3.18
-2.21

C

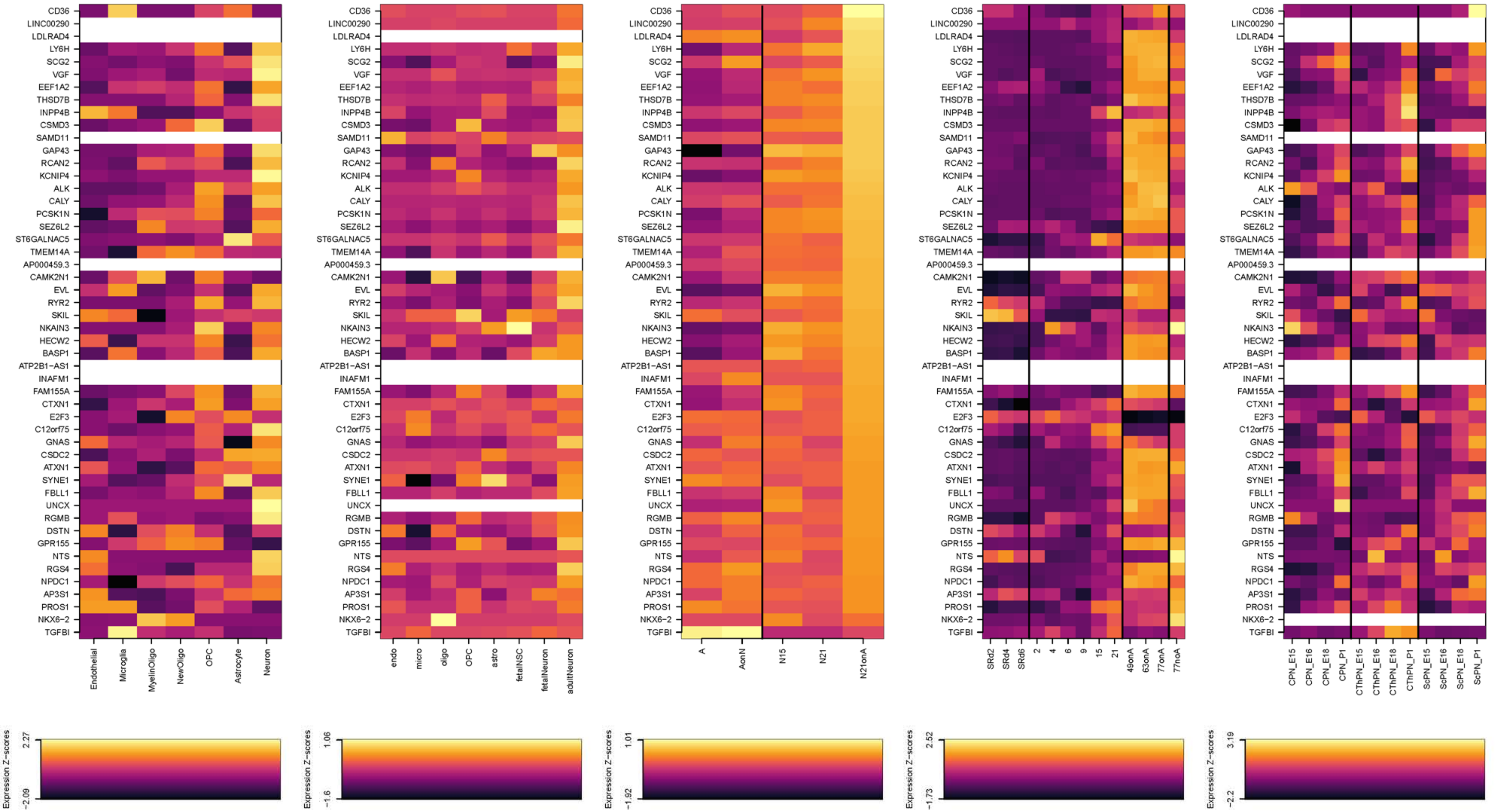
Zhang et al 2014
Darmanis et al 2015
This report: DEG.NvNA.NAup
Burke et al 2020
Molyneaux et al 2015 (DeCon)
CD36
LINC00290
LDLRAD4
LY6H
SCG2
VGF
EEF1A2
THSD7B
INPP4B
CSMD3
SAMD11
GAP43
RCAN2
KCNIP4
ALK
CALY
PCSK1N
SEZ6L2
ST6GALNAC5
TMEM14A
AP000459.3
CAMK2N1
EVL
RYR2
SKIL
NKAIN3
HECW2
BASP1
ATP2B1-AS1
INAFM1
FAM155A
CTXN1
E2F3
C12orf75
GNAS
CSDC2
ATXN1
SYNE1
FBLL1
UNCX
RGMB
DSTN
GPR155
NTS
RGS4
NPDC1
AP3S1
PROS1
NKX6-2
TGFBI
Endothelial
Microglia
MyelinOligo
NewOligo
OPC
Astrocyte
Neuron
endo
micro
oligo
OPC
astro
fetalNSC
fetalNeuron
adultNeuron
A
AonN
N15
N21
N21onA
SRd2
SRd4
SRd6
2
4
6
9
15
21
49onA
63onA
77onA
77noA
CPN_E15
CPN_E16
CPN_E18
CPN_P1
CThPN_E15
CThPN_E16
CThPN_E18
CThPN_P1
ScPN_E15
ScPN_E16
ScPN_E18
ScPN_P1
Expression Z-scores
2.27
-2.09
1.06
-1.6
1.01
-1.92
2.52
-1.73
3.19
-2.2

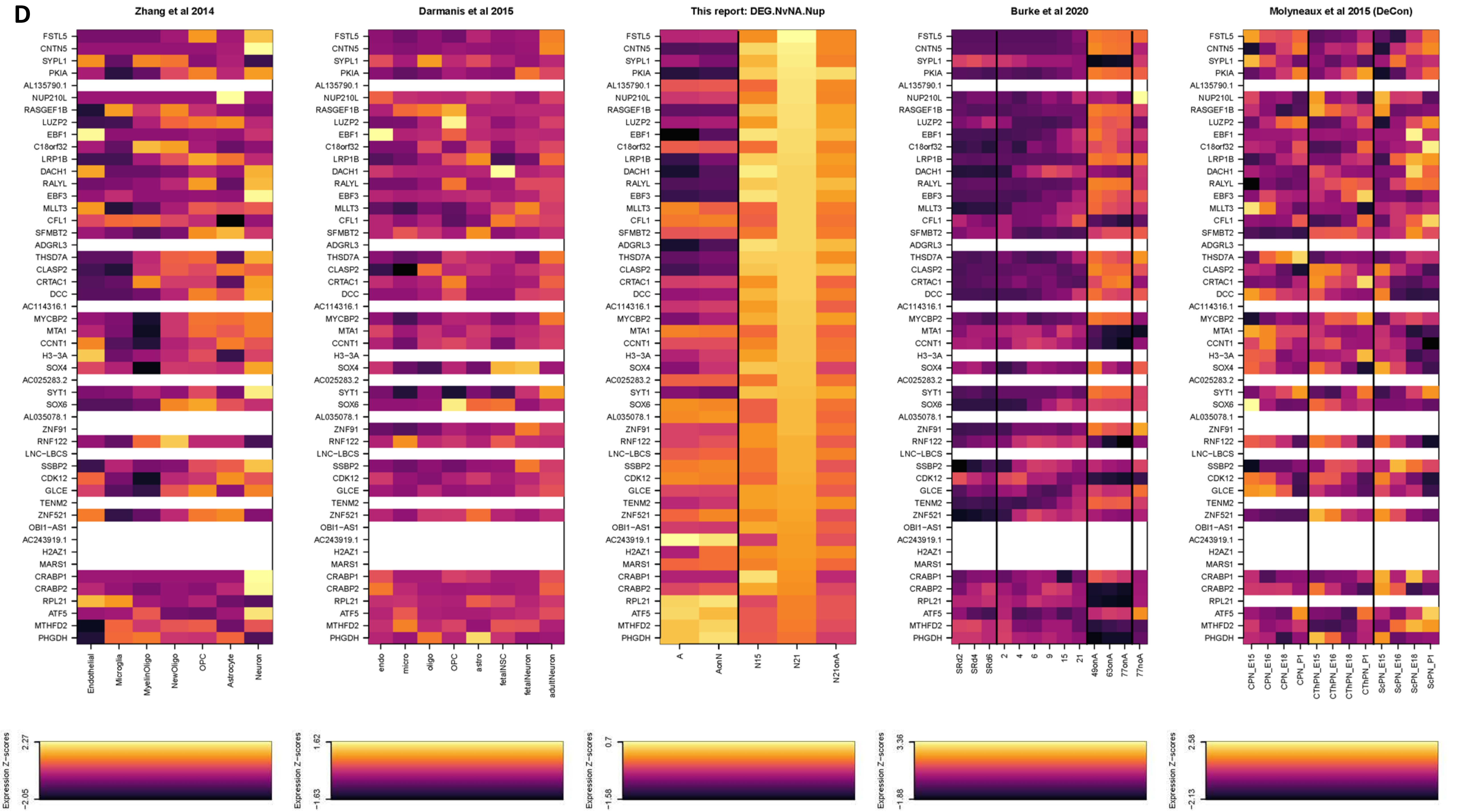
D
Zhang et al 2014
Darmanis et al 2015
This report: DEG.NvNA.Nup
Burke et al 2020
Molyneaux et al 2015 (DeCon)
FSTL5
CNTN5
SYPL1
PKIA
AL135790.1
NUP210L
RASGEF1B
LUZP2
EBF1
C18orf32
LRP1B
DACH1
RALYL
EBF3
MLLT3
CFL1
SFMBT2
ADGRL3
THSD7A
CLASP2
CRTAC1
DCC
AC114316.1
MYCBP2
MTA1
CCNT1
H3-3A
SOX4
AC025283.2
SYT1
SOX6
AL035078.1
ZNF91
RNF122
LNC-LBCS
SSBP2
CDK12
GLCE
TENM2
ZNF521
OBI1-AS1
AC243919.1
H2AZ1
MARS1
CRABP1
CRABP2
RPL21
ATF5
MTHFD2
PHGDH
Endothelial
Microglia
MyelinOligo
NewOligo
OPC
Astrocyte
Neuron
endo
micro
oligo
OPC
astro
fetalNSC
fetalNeuron
adultNeuron
A
AonN
N15
N21
N21onA
SRd2
SRd4
SRd6
2
4
6
9
15
21
49onA
63onA
77onA
77noA
CPN_E15
CPN_E16
CPN_E18
CPN_P1
CThPN_E15
CThPN_E16
CThPN_E18
CThPN_P1
ScPN_E15
ScPN_E16
ScPN_E18
ScPN_P1
Expression Z-scores
-2.05
2.27
-1.63
1.62
-1.58
0.7
-1.88
3.36
-2.13
2.58

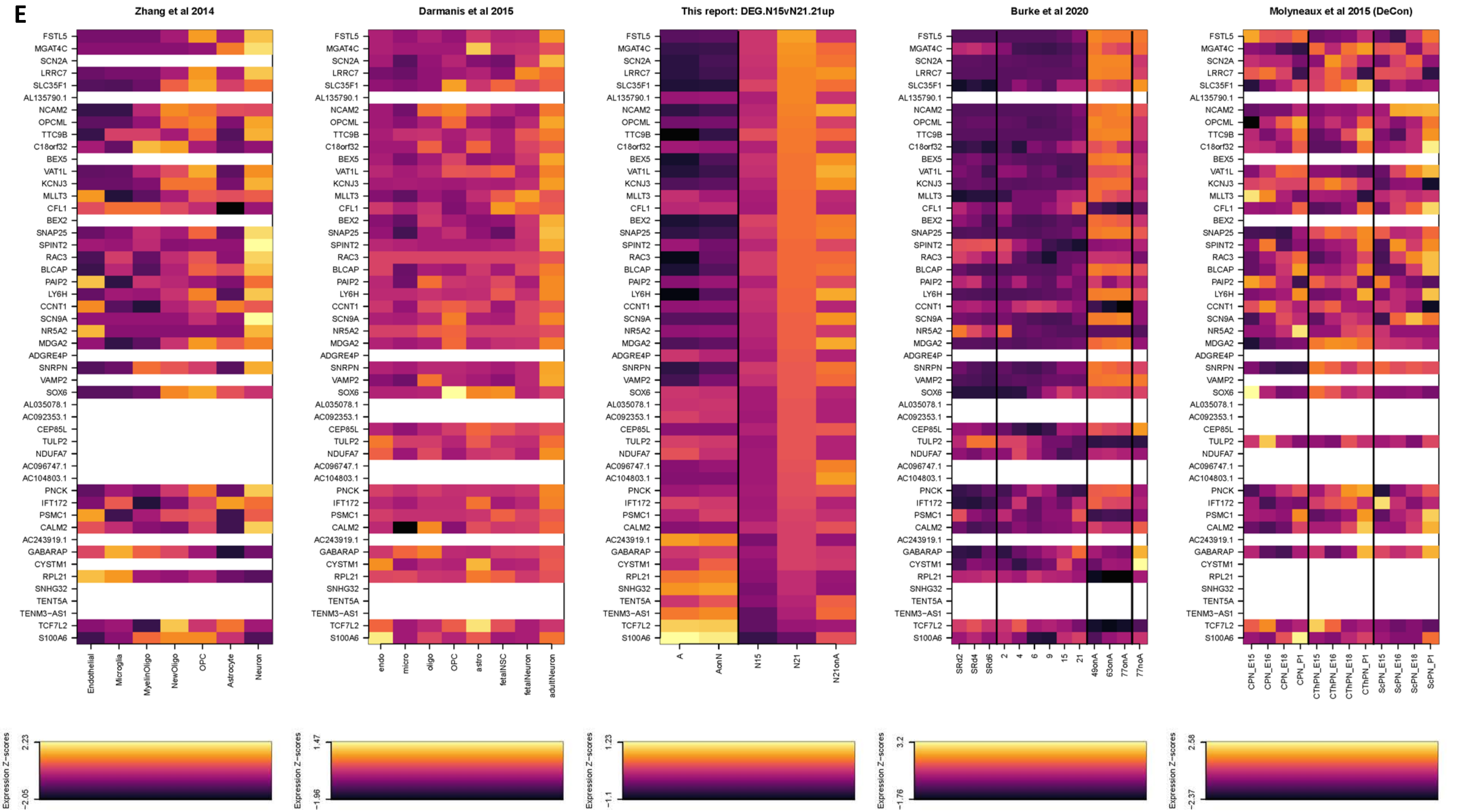
E
Zhang et al 2014
Darmanis et al 2015
This report: DEG.N15vN21.21up
Burke et al 2020
Molyneaux et al 2015 (DeCon)
FSTL5
MGAT4C
SCN2A
LRRC7
SLC35F1
AL135790.1
NCAM2
OPCML
TTC9B
C18orf32
BEX5
VAT1L
KCNJ3
MLLT3
CFL1
BEX2
SNAP25
SPINT2
RAC3
BLCAP
PAIP2
LY6H
CCNT1
SCN9A
NR5A2
MDGA2
ADGRE4P
SNRPN
VAMP2
SOX6
AL035078.1
AC092353.1
CEP85L
TULP2
NDUFA7
AC096747.1
AC104803.1
PNCK
IFT172
PSMC1
CALM2
AC243919.1
GABARAP
CYSTM1
RPL21
SNHG32
TENT5A
TENM3-AS1
TCF7L2
S100A6
Endothelial
Microglia
MyelinOligo
NewOligo
OPC
Astrocyte
Neuron
endo
micro
oligo
OPC
astro
fetalNSC
fetalNeuron
adultNeuron
A
AonN
N15
N21
N21onA
SRd2
SRd4
SRd6
2
4
6
9
15
21
49onA
63onA
77onA
77noA
CPN_E15
CPN_E16
CPN_E18
CPN_P1
CThPN_E15
CThPN_E16
CThPN_E18
CThPN_P1
ScPN_E15
ScPN_E16
ScPN_E18
ScPN_P1
Expression Z-scores
2.23
-2.05
1.47
-1.96
1.23
-1.1
3.2
-1.76
2.58
-2.37

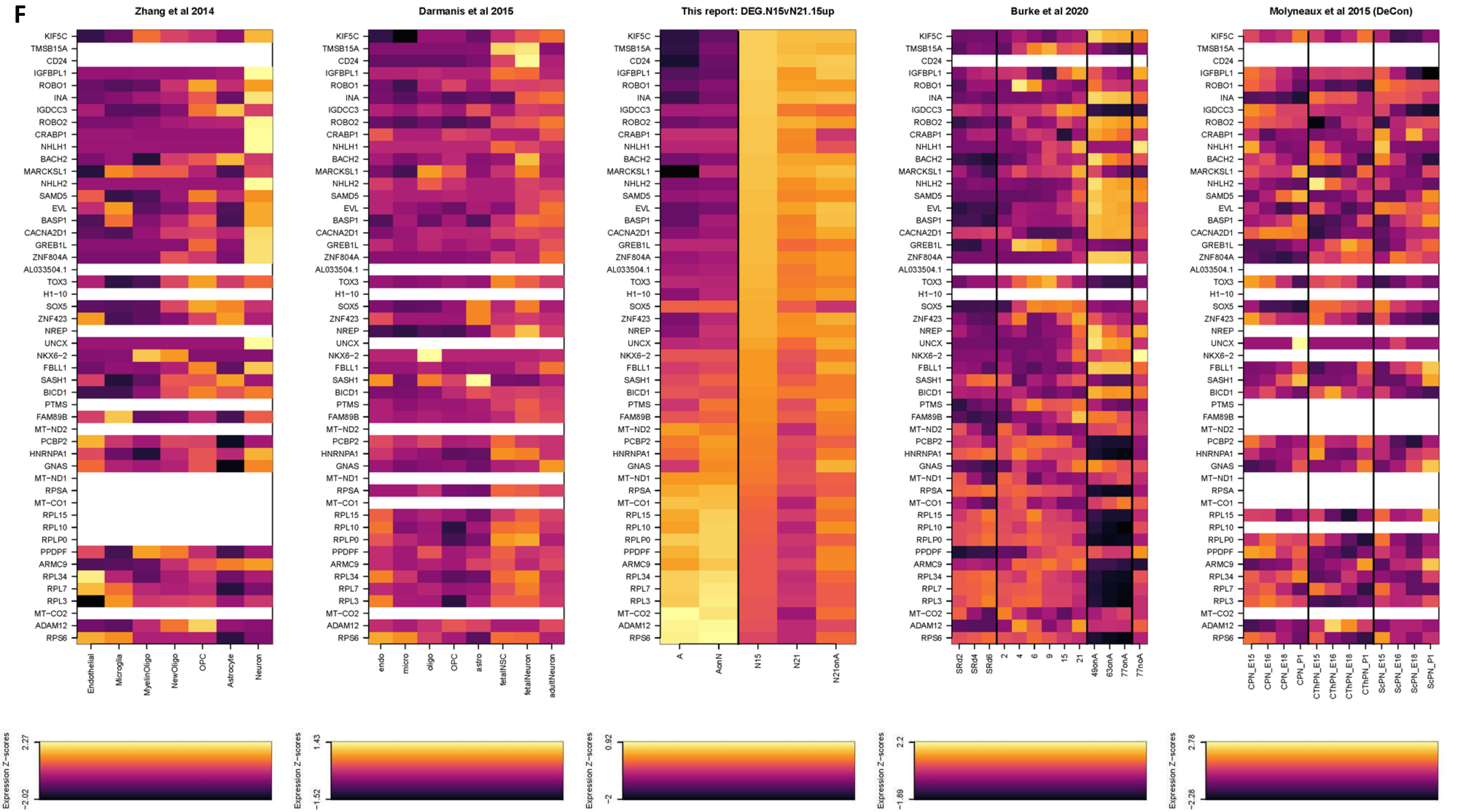
F
Zhang et al 2014
Darmanis et al 2015
This report: DEG.N15vN21.15up
Burke et al 2020
Molyneaux et al 2015 (DeCon)
KIF5C
TMSB15A
CD24
IGFBPL1
ROBO1
INA
IGDCC3
ROBO2
CRABP1
NHLH1
BACH2
MARCKSL1
NHLH2
SAMD5
EVL
BASP1
CACNA2D1
GREB1L
ZNF804A
AL033504.1
TOX3
H1−10
SOX5
ZNF423
NREP
UNCX
NKX6−2
FBLL1
SASH1
BICD1
PTMS
FAM89B
MT−ND2
PCBP2
HNRNPA1
GNAS
MT−ND1
RPSA
MT−CO1
RPL15
RPL10
RPLP0
PPDPF
ARMC9
RPL34
RPL7
RPL3
MT−CO2
ADAM12
RPS6
Endothelial
Microglia
MyelinOligo
NewOligo
OPC
Astrocyte
Neuron
endo
micro
oligo
OPC
astro
fetalNSC
fetalNeuron
adultNeuron
A
AonN
N15
N21
N21onA
SRd2
SRd4
SRd6
2
4
6
9
15
21
49onA
63onA
77onA
77noA
CPN_E15
CPN_E16
CPN_E18
CPN_P1
CThPN_E15
CThPN_E16
CThPN_E18
CThPN_P1
ScPN_E15
ScPN_E16
ScPN_E18
ScPN_P1
Expression Z−scores
2.27
−2.02
Expression Z−scores
1.43
−1.52
Expression Z−scores
0.92
−2
Expression Z−scores
2.2
−1.89
Expression Z−scores
2.78
−2.28

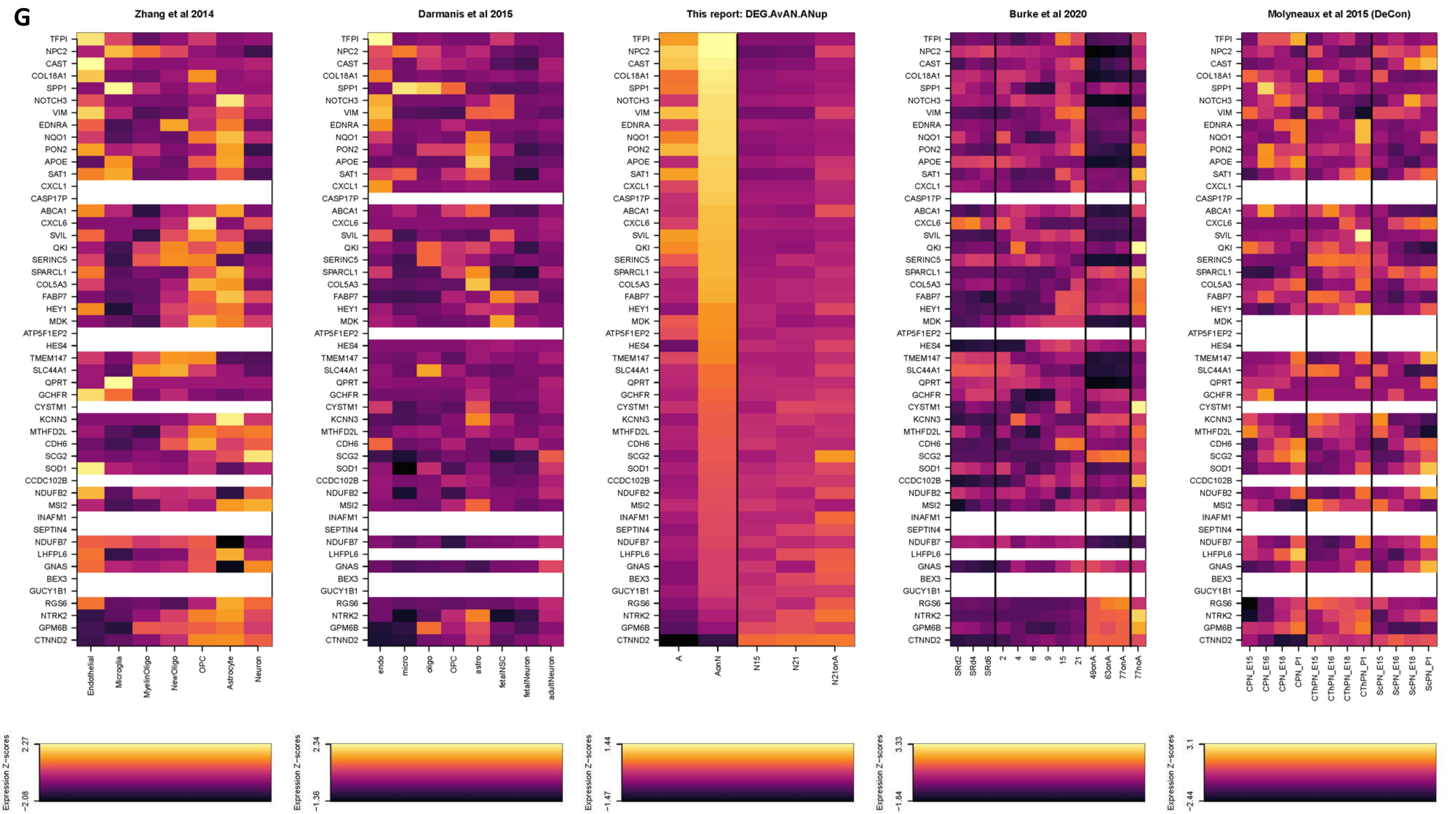
G
Zhang et al 2014
TFPI
NPC2
CAST
COL18A1
SPP1
NOTCH3
VIM
EDNRA
NQO1
PON2
APOE
SAT1
CXCL1
CASP17P
ABCA1
CXCL6
SVIL
QKI
SERINC5
SPARCL1
COL5A3
FABP7
HEY1
MDK
ATP5F1EP2
HES4
TMEM147
SLC44A1
QPRT
GCHFR
CYSTM1
KCNN3
MTHFD2L
CDH6
SCG2
SOD1
CCDC102B
NDUFB2
MSI2
INAFM1
SEPTIN4
NDUFB7
LHFPL6
GNAS
BEX3
GUCY1B1
RGS6
NTRK2
GPM6B
CTNND2
Endothelial
Microglia
MyelinOligo
NewOligo
OPC
Astrocyte
Neuron
Expression Z-scores
2.27
-2.08
Darmanis et al 2015
TFPI
NPC2
CAST
COL18A1
SPP1
NOTCH3
VIM
EDNRA
NQO1
PON2
APOE
SAT1
CXCL1
CASP17P
ABCA1
CXCL6
SVIL
QKI
SERINC5
SPARCL1
COL5A3
FABP7
HEY1
MDK
ATP5F1EP2
HES4
TMEM147
SLC44A1
QPRT
GCHFR
CYSTM1
KCNN3
MTHFD2L
CDH6
SCG2
SOD1
CCDC102B
NDUFB2
MSI2
INAFM1
SEPTIN4
NDUFB7
LHFPL6
GNAS
BEX3
GUCY1B1
RGS6
NTRK2
GPM6B
CTNND2
endo
micro
oligo
OPC
astro
fetalNSC
fetalNeuron
adultNeuron
Expression Z-scores
2.34
-1.36
This report: DEG.AvAN.ANup
TFPI
NPC2
CAST
COL18A1
SPP1
NOTCH3
VIM
EDNRA
NQO1
PON2
APOE
SAT1
CXCL1
CASP17P
ABCA1
CXCL6
SVIL
QKI
SERINC5
SPARCL1
COL5A3
FABP7
HEY1
MDK
ATP5F1EP2
HES4
TMEM147
SLC44A1
QPRT
GCHFR
CYSTM1
KCNN3
MTHFD2L
CDH6
SCG2
SOD1
CCDC102B
NDUFB2
MSI2
INAFM1
SEPTIN4
NDUFB7
LHFPL6
GNAS
BEX3
GUCY1B1
RGS6
NTRK2
GPM6B
CTNND2
A
AonN
N15
N21
N21onA
Expression Z-scores
1.44
-1.47
Burke et al 2020
TFPI
NPC2
CAST
COL18A1
SPP1
NOTCH3
VIM
EDNRA
NQO1
PON2
APOE
SAT1
CXCL1
CASP17P
ABCA1
CXCL6
SVIL
QKI
SERINC5
SPARCL1
COL5A3
FABP7
HEY1
MDK
ATP5F1EP2
HES4
TMEM147
SLC44A1
QPRT
GCHFR
CYSTM1
KCNN3
MTHFD2L
CDH6
SCG2
SOD1
CCDC102B
NDUFB2
MSI2
INAFM1
SEPTIN4
NDUFB7
LHFPL6
GNAS
BEX3
GUCY1B1
RGS6
NTRK2
GPM6B
CTNND2
SRd2
SRd4
SRd6
2
4
6
9
15
21
49onA
63onA
77onA
77noA
Expression Z-scores
3.33
-1.84
Molyneaux et al 2015 (DeCon)
TFPI
NPC2
CAST
COL18A1
SPP1
NOTCH3
VIM
EDNRA
NQO1
PON2
APOE
SAT1
CXCL1
CASP17P
ABCA1
CXCL6
SVIL
QKI
SERINC5
SPARCL1
COL5A3
FABP7
HEY1
MDK
ATP5F1EP2
HES4
TMEM147
SLC44A1
QPRT
GCHFR
CYSTM1
KCNN3
MTHFD2L
CDH6
SCG2
SOD1
CCDC102B
NDUFB2
MSI2
INAFM1
SEPTIN4
NDUFB7
LHFPL6
GNAS
BEX3
GUCY1B1
RGS6
NTRK2
GPM6B
CTNND2
CPN_E15
CPN_E16
CPN_E18
CPN_P1
CThPN_E15
CThPN_E16
CThPN_E18
CThPN_P1
ScPN_E15
ScPN_E16
ScPN_E18
ScPN_P1
Expression Z-scores
3.1
-2.44

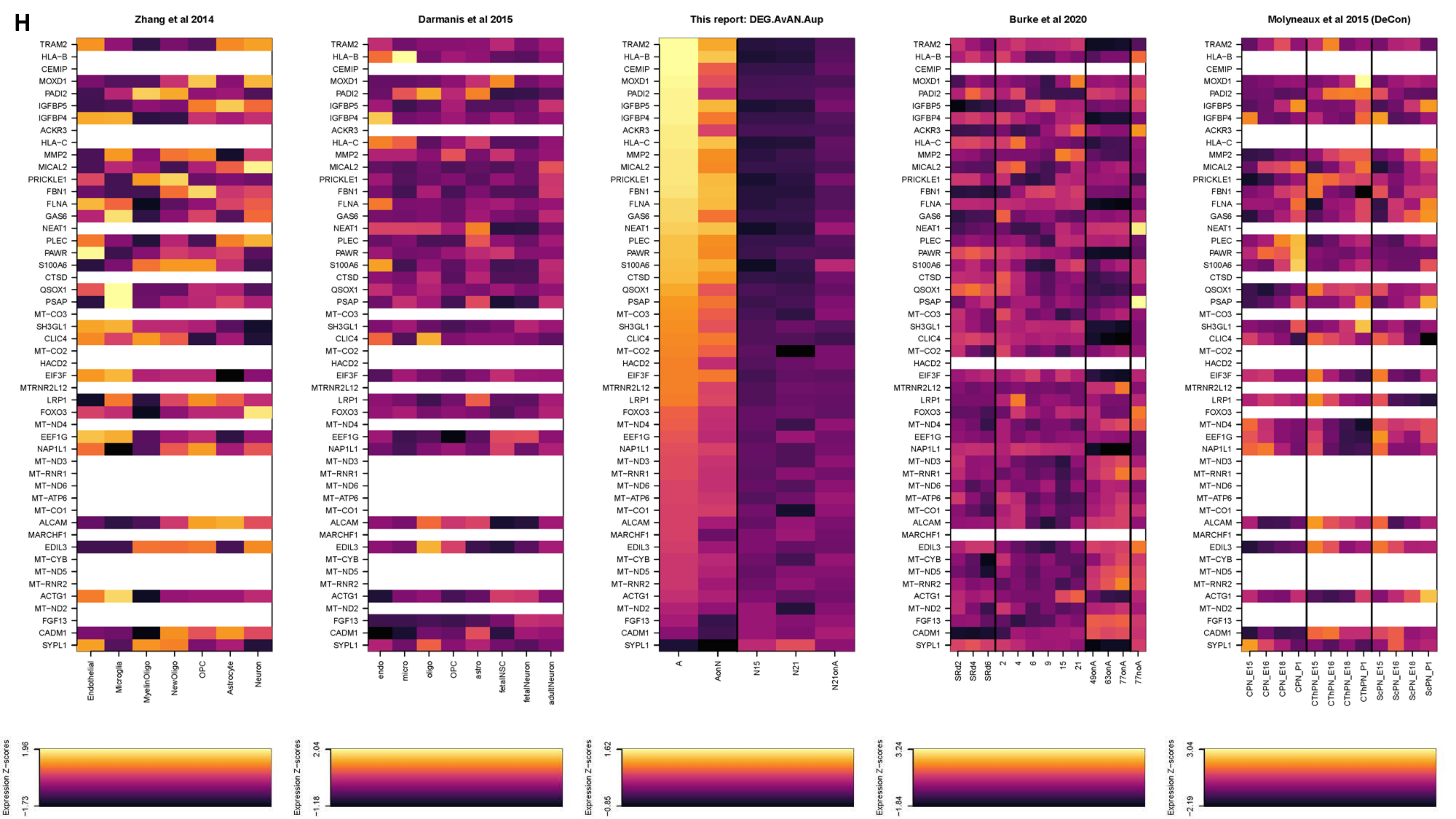
H
Zhang et al 2014
TRAM2
HLA-B
CEMIP
MOXD1
PADI2
IGFBP5
IGFBP4
ACKR3
HLA-C
MMP2
MICAL2
PRICKLE1
FBN1
FLNA
GAS6
NEAT1
PLEC
PAWR
S100A6
CTSD
QSOX1
PSAP
MT-CO3
SH3GL1
CLIC4
MT-CO2
HACD2
EIF3F
MTRNR2L12
LRP1
FOXO3
MT-ND4
EEF1G
NAP1L1
MT-ND3
MT-RNR1
MT-ND6
MT-ATP6
MT-CO1
ALCAM
MARCHF1
EDIL3
MT-CYB
MT-ND5
MT-RNR2
ACTG1
MT-ND2
FGF13
CADM1
SYPL1
Endothelial
Microglia
MyelinOligo
NewOligo
OPC
Astrocyte
Neuron
Expression Z-scores
1.96
-1.73
Darmanis et al 2015
TRAM2
HLA-B
CEMIP
MOXD1
PADI2
IGFBP5
IGFBP4
ACKR3
HLA-C
MMP2
MICAL2
PRICKLE1
FBN1
FLNA
GAS6
NEAT1
PLEC
PAWR
S100A6
CTSD
QSOX1
PSAP
MT-CO3
SH3GL1
CLIC4
MT-CO2
HACD2
EIF3F
MTRNR2L12
LRP1
FOXO3
MT-ND4
EEF1G
NAP1L1
MT-ND3
MT-RNR1
MT-ND6
MT-ATP6
MT-CO1
ALCAM
MARCHF1
EDIL3
MT-CYB
MT-ND5
MT-RNR2
ACTG1
MT-ND2
FGF13
CADM1
SYPL1
endo
micro
oligo
OPC
astro
fetalNSC
fetalNeuron
adultNeuron
Expression Z-scores
2.04
-1.18
This report: DEG.AvAN.Aup
TRAM2
HLA-B
CEMIP
MOXD1
PADI2
IGFBP5
IGFBP4
ACKR3
HLA-C
MMP2
MICAL2
PRICKLE1
FBN1
FLNA
GAS6
NEAT1
PLEC
PAWR
S100A6
CTSD
QSOX1
PSAP
MT-CO3
SH3GL1
CLIC4
MT-CO2
HACD2
EIF3F
MTRNR2L12
LRP1
FOXO3
MT-ND4
EEF1G
NAP1L1
MT-ND3
MT-RNR1
MT-ND6
MT-ATP6
MT-CO1
ALCAM
MARCHF1
EDIL3
MT-CYB
MT-ND5
MT-RNR2
ACTG1
MT-ND2
FGF13
CADM1
SYPL1
A
AonN
N15
N21
N21onA
Expression Z-scores
1.62
-0.85
Burke et al 2020
TRAM2
HLA-B
CEMIP
MOXD1
PADI2
IGFBP5
IGFBP4
ACKR3
HLA-C
MMP2
MICAL2
PRICKLE1
FBN1
FLNA
GAS6
NEAT1
PLEC
PAWR
S100A6
CTSD
QSOX1
PSAP
MT-CO3
SH3GL1
CLIC4
MT-CO2
HACD2
EIF3F
MTRNR2L12
LRP1
FOXO3
MT-ND4
EEF1G
NAP1L1
MT-ND3
MT-RNR1
MT-ND6
MT-ATP6
MT-CO1
ALCAM
MARCHF1
EDIL3
MT-CYB
MT-ND5
MT-RNR2
ACTG1
MT-ND2
FGF13
CADM1
SYPL1
SRd2
SRd4
SRd6
2
4
6
9
15
21
49onA
63onA
77onA
77noA
Expression Z-scores
3.24
-1.84
Molyneaux et al 2015 (DeCon)
TRAM2
HLA-B
CEMIP
MOXD1
PADI2
IGFBP5
IGFBP4
ACKR3
HLA-C
MMP2
MICAL2
PRICKLE1
FBN1
FLNA
GAS6
NEAT1
PLEC
PAWR
S100A6
CTSD
QSOX1
PSAP
MT-CO3
SH3GL1
CLIC4
MT-CO2
HACD2
EIF3F
MTRNR2L12
LRP1
FOXO3
MT-ND4
EEF1G
NAP1L1
MT-ND3
MT-RNR1
MT-ND6
MT-ATP6
MT-CO1
ALCAM
MARCHF1
EDIL3
MT-CYB
MT-ND5
MT-RNR2
ACTG1
MT-ND2
FGF13
CADM1
SYPL1
CPN_E15
CPN_E16
CPN_E18
CPN_P1
CThPN_E15
CThPN_E16
CThPN_E18
CThPN_P1
ScPN_E15
ScPN_E16
ScPN_E18
ScPN_P1
Expression Z-scores
3.04
-2.19
